# Molecular Glue Degraders Enhance CAPRIN1‐Dependent Lysosomal Degradation of APP and Reduce Amyloid β in Alzheimer's Disease

**DOI:** 10.1002/advs.77575

**Published:** 2026-09-01

**Authors:** Sunghan Jung, Xu Wang, Bin Liu, Raktim Roy, Nancy Jaiswal, Ratan K. Rai, Yuichiro Takagi, Cen Gao, Lifan Zeng, Ho‐Yin Lo, Reagan K. Wohlford, Ryan K. Higgins, Bruce T. Lamb, Anantha Shekhar, Anita C. Bellail, Chunhai Hao

**Affiliations:** ^1^ Department of Pathology and Laboratory Medicine Indiana University School of Medicine Indianapolis Indiana USA; ^2^ Department of Biochemistry and Molecular Biology Indiana University School of Medicine Indianapolis Indiana USA; ^3^ Synovel Laboratory LLC Danbury Connecticut USA; ^4^ Department of Medical and Molecular Genetics Indiana University School of Medicine Indianapolis Indiana USA; ^5^ Stark Neurosciences Research Institute Indiana University School of Medicine Indianapolis Indiana USA; ^6^ Department of Psychiatry University of Pittsburgh School of Medicine Pittsburgh Pennsylvania USA; ^7^ Degrome Therapeutics, Inc. Indianapolis Indiana USA; ^8^ Indiana University Melvin and Bren Simon Comprehensive Cancer Center Indianapolis Indiana USA

**Keywords:** Alzheimer's disease, amyloid precursor protein, amyloid β plaque, induced pluripotent stem cell, molecule glue degrader

## Abstract

The amyloid cascade involving the production and deposition of amyloid β (Aβ) peptides derived from amyloid precursor protein (APP) is a leading hypothesis in the pathogenesis of Alzheimer's disease (AD). However, disease‐modifying therapies targeting this cascade remain an unmet challenge. Here, we identify small molecules that degrade APP and reduce Aβ production through a targeted protein degradation strategy. Using genetically engineered APP cell models and induced pluripotent stem cell (iPSC)‐derived neurons from patients with AD, we demonstrate that cytoplasmic activation/proliferation‐associated protein 1 (CAPRIN1) is expressed in neurons and physically interacts with APP. Screening of a CAPRIN1‐targeted compound library identified compound 0043, which promotes intracellular APP degradation and reduces extracellular Aβ release. Structure–activity relationship optimization of 0043 derivatives yielded compound 0152 with improved potency and blood–brain barrier permeability. In AD iPSC‐derived neurons, these compounds enhance CAPRIN1–APP interactions and promote CAPRIN1‐dependent APP degradation through the endolysosomal pathway. CAPRIN1 is predominantly expressed in neurons in AD postmortem brains and iPSC‐derived brain organoids. Systemic administration of compound 0152 significantly reduced APP levels, Aβ production, and amyloid burden in 5xFAD mice. These findings establish CAPRIN1‐dependent, lysosome‐targeted molecular glue degraders as a potential APP‐targeted therapeutic strategy for AD.

## Graphical abstract

A new class of lysosome‐directed molecular glue degraders selectively enhance CAPRIN1–APP interactions, driving APP degradation and reducing amyloid‐β production in human neurons and Alzheimer's disease mouse models. This CAPRIN1‐dependent targeted protein degradation strategy reveals a previously unrecognized therapeutic approach for disrupting the amyloid cascade in Alzheimer's disease.

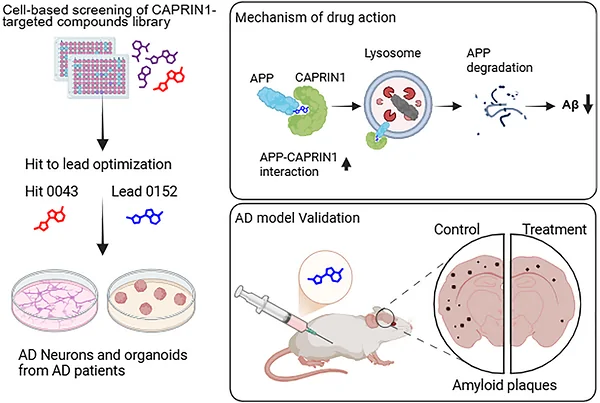

## 1 Introduction

Alzheimer's disease (AD) is a progressive neurodegenerative disorder and the leading cause of dementia worldwide [1]. AD is characterized by the extracellular accumulation of amyloid‐β (Aβ) peptides and the intracellular aggregation of hyperphosphorylated tau, predominantly within the cerebral cortex and hippocampus [2]. Aβ peptides are generated via the sequential cleavage of amyloid precursor protein (APP) by β‐secretase 1 (BACE1) and γ‐secretase in neurons, followed by their extracellular release and aggregation into plaques, which represents the initial event that drives downstream tau pathology, neuroinflammation, and neuronal loss [3]. The majority of Aβ has a length of 40 residues (Aβ40), but a small fraction of the longer Aβ42 is highly hydrophobic and prone to the accumulation and formation of extracellular plaques [4]. Therapeutic strategies aimed at interrupting this APP‐amyloid cascade have led to the development of several disease‐modifying agents, including BACE1 and γ‐secretase inhibitors and monoclonal antibodies (mAbs) targeting Aβ [3]. While secretase inhibitors have failed to show clinical efficacy, mAbs such as aducanumab, lecanemab, and donanemab have received FDA approval for patients with early‐stage AD, underscoring the therapeutic potential of targeting APP‐derived Aβ amyloids [5, 6, 7].

Complex genomic and molecular alterations contribute to dysregulated Aβ production and clearance, which critically influence the onset and progression of AD and related dementias (ADRD) [8]. In Down syndrome (DS), trisomy 21 leads to APP gene triplication, resulting in elevated APP expression and Aβ production, which drive ADRD that typically appears before age 40 [9]. Similarly, early‐onset familial AD (fAD), which manifests before age 65, is driven by mutations in *APP*, *Presenilin‐1* (*PSEN1*) or *PSEN2* that promote increased APP expression and generation of aggregation‐prone Aβ42 peptides [8]. Late‐onset sporadic AD (sAD), which arises after age 60, is multifactorial and involves mechanisms such as neuronal genomic mosaicism and RNA dysregulation, both contributing to neuronal APP expression and Aβ accumulation [10]. The APOE4 allele (ε4) increases Alzheimer's risk by reducing Aβ42 clearance through impaired microglia functions [11]. These observations highlight APP‐derived Aβ amyloids as compelling therapeutic targets for AD and ADRD.

Targeted protein degradation (TPD) is an emerging therapeutic paradigm that shifts drug discovery from functional inhibition to selective degradation of targeted proteins by harnessing either proteasomal or lysosomal degradation pathways [12]. Proteasome‐directed degraders developed to date primarily comprise two major modalities: monovalent molecular glue degraders and heterobifunctional proteolysis‐targeting chimeras (PROTACs) [13]. The discovery of thalidomide‐ and sulfonamide‐derivatives demonstrated that monovalent small molecules could bind the E3 ligase cereblon (CRBN)‐cullin 4 (CUL4) [14, 15, 16, 17] and DCAF15‐CUL4 [18, 19, 20], to induce or stabilize interaction with neosubstrate proteins and promote protein ubiquitination and proteasomal degradation. PROTACs were subsequently developed as heterobifunctional molecules consisting of a ligand for an E3 ligase and a ligand for a target protein connected by a chemical linker, enabling induced proximity, ubiquitination, and proteasomal degradation of targeted proteins [21]. More recently, lysosome‐directed degraders, such as large lysosome‐targeting‐chimeras (LYTACs), have been developed to engage lysosome‐targeting receptors and deliver membrane‐associated proteins to the lysosomal system for degradation [22, 23]. Here, we report compounds that bind at the interface between amyloid precursor protein (APP) and cytoplasmic activation/proliferation‐associated protein 1 (CAPRIN1), stabilize their interaction, and promote CAPRIN1‐mediated lysosomal degradation of the membrane‐bound APP. Classical molecular glue degraders stabilize interactions between E3 ligases and substrate proteins to promote proteasomal degradation. In contrast, our compounds stabilize the interaction between CAPRIN1 and APP, thereby promoting lysosomal degradation of APP without engaging an E3 ligase. These findings define a new mechanistic subclass of lysosome‐directed molecular glue degraders that extends the molecular glue paradigm beyond the canonical ubiquitin–proteasome pathway to lysosomal degradation.

CAPRIN1 is an RNA‐binding protein [24] that is highly expressed in neurons, where it regulates the transport and translation of mRNAs encoding synaptic proteins [25]. CAPRIN1 deficiency has been associated with autism spectrum disorders and long‐term memory impairment [26, 27]. CAPRIN1 has previously been identified as an APP‐binding partner [28], but its functional role in APP homeostasis remains poorly understood. Here, we demonstrate that CAPRIN1 is predominantly expressed in neurons, where it interacts with APP and mediates its trafficking to the endo‐lysosomal pathway for degradation. Furthermore, we identify molecular glue degraders that bind the interface between CAPRIN1 and APP, enhancing the CAPRIN1–APP interaction and thereby promoting CAPRIN1‐mediated lysosomal degradation of APP in human neurons derived from induced pluripotent stem cells (iPSCs) from AD patients. Mechanistically, CAPRIN1 exhibits dual functional domains, with its C‐terminal region mediating mRNA binding, whereas its N‐terminal region engages the degraders, thereby preserving the neuronal functions of cytosolic CAPRIN1. This mechanism significantly reduces Aβ42 accumulation and plaque deposition in AD mouse brains, revealing a previously unrecognized lysosome‐directed molecular glue strategy and a potential therapeutic approach for AD.

## 2 Results

### 2.1 Discovery of Active Compounds as APP Degraders Through Cell‐Based Screening

We previously reported CAPRIN1‐targeted molecular degraders that bind to CAPRIN1 and F‐box protein 42 (FBXO42), promoting FBXO42‐mediated proteasomal degradation of small ubiquitin‐related modifier 1 (SUMO1), an oncoprotein, in human cancers [29]. CAPRIN1 is a neuronal RNA‐binding protein involved in neuronal development and neurodegeneration [27]. CAPRIN1 was reported to be a binding partner of APP [28]. Therefore, we thought to investigate whether CAPRIN1‐targeted degraders could promote APP degradation in APP‐expressing cell models. To establish screening models, we tagged wild‐type APP695 (APP695), the predominant neuronal isoform, with the small HiBiT tag (APP695‐HiBiT) and expressed APP695‐HiBiT in HEK293 cells. Cell lysates were analyzed using a luminescence‐based assay of HiBiT‐tagged protein [30], identifying clone #3 and #9 with robust APP695‐HiBiT expression (Figure S1A). Western blotting and ELISA confirmed intracellular expression of APP695 and extracellular release of Aβ42 in these clones (Figure S1B,C).

**FIGURE 1:**
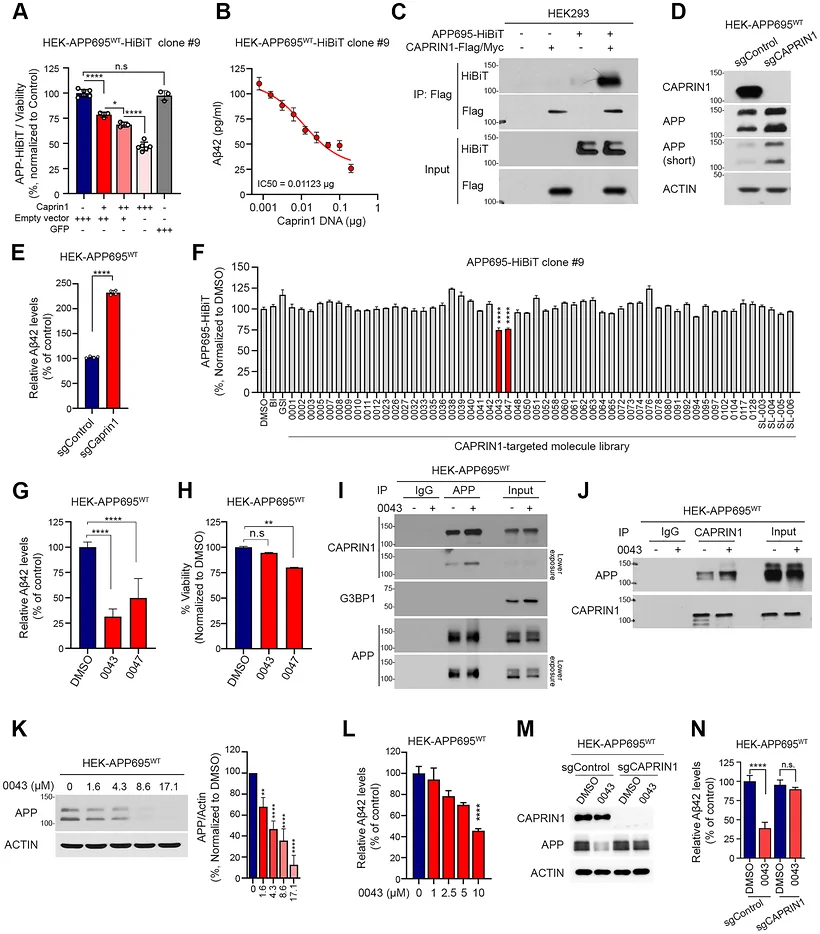
CAPRIN1 regulates APP levels and identifies compound 0043 as an APP degrader. (A) APP‐HiBiT levels in HEK293 cells transfected with increasing amounts of CAPRIN1 expression plasmid (+ = 50 ng, ++ = 100 ng, +++ = 200 ng). Total DNA was kept constant using an empty vector. GFP (200 ng) was included as an unrelated control. (B) Dose–response curve for Amyloid‐β42 (Aβ42) levels upon CAPRIN1 overexpression with calculated IC_50_ (mean ± s.d.). (C) In HEK cells co‐expressing APP695‐HiBiT and Flag‐tagged CAPRIN1, CAPRIN1 was Co‐immunoprecipitated (Co‐IP) and APP was detected by immunoblot. (D) Western blot (WB) analysis of APP and CAPRIN1 expression in sgCAPRIN1 HEK‐APP695^WT^ cells. (E) Relative Aβ42 levels in conditioned media measured by ELISA in sgCAPRIN1 vs. sgControl HEK‐APP695^WT^ cells (mean ± s.d.). (F) HiBiT signal in APP695‐HiBiT clone #9 cells treated for 24 h with 10 uM CAPRIN1‐targeted small‐molecule library (normalized to the DMSO control). Compounds 0043 and 0047 are highlighted in red. (G, H) HEK‐APP695^WT^ cells treated with DMSO, 0043 or 0047 (10 um) for 72 h. Relative Aβ42 levels in conditioned media were measured by ELISA (G), and cell viability was assessed using CellTiter‐Glo assay (H) (mean ± s.d.; *n* = 3; ^**^ *p* < 0.01, ^****^ *p* < 0.0001; one‐way ANOVA). (I, J) Co‐IP showing enhanced APP‐CAPRIN1 interaction in HEK‐APP695^WT^ cells treated with 0043 (10 µm, 24 h). APP was immunoprecipitated and CAPRIN1 detected in (I), whereas CAPRIN1 was immunoprecipitated and APP detected in (J). (K) Western blot of APP in HEK‐APP695^WT^ cells treated with increasing concentrations of 0043 for 72 h. (L) Relative Aβ42 levels in conditioned media after 72 h treatment with 0043 (mean ± s.d.; *n* = 3; ^****^ *p* < 0.0001). (M) Western blot showing APP and CAPRIN1 expression in sgControl and sgCAPRIN1 HEK‐APP695^WT^ cells treated with DMSO or 0043 (10 µm, 72 h). (N) Relative Aβ42 levels in conditioned media of the same cells as in (M) after 72 h (mean ± s.d.; *n* = 3; ^****^ *p* < 0.0001; one‐way ANOVA with Tukey's post hoc test). Aβ42 levels in conditioned media were normalized to the DMSO control.

To assess CAPRIN1's role in APP regulation, *CAPRIN1* DNA was overexpressed in HEK‐APP695‐HiBiT clones, leading to a dose‐dependent decrease in intracellular APP and extracellular Aβ42 (Figure 1A,B). APP is a glycoprotein that undergoes both N‐ and O‐linked glycosylation during its maturation, resulting in the lower band corresponds to the immature (core‐glycosylated) form and the upper band represents the mature (fully glycosylated) form of APP [31]. Co‐transfection of HiBiT‐APP695 and Flag‐tagged CAPRIN1 in HEK cells, followed by Flag immunoprecipitation and immunoblotting with HiBiT antibodies, demonstrated an interaction between CAPRIN1 and APP (Figure 1C). Next, we generated a CAPRIN1 knockout (KO) by CRISPR gene editing following our protocol [29]. In brief, sgRNAs targeting CAPRIN1 were cloned into a lentiCRISPRv2 vector and introduced into cells via lentiviral transduction, resulting in gene disruption through non‐homologous end joining rather than large genomic deletion. Following transduction, single‐cell clones were isolated and expanded, and CAPRIN1 KO efficiency was confirmed by western blotting. Multiple independent clones were screened by western blotting for CAPRIN1 KO, and 2 independent KO clones were used for the experiments to further define the role of CAPRIN1 in APP expression. The results showed that CRISPR‐Cas9 KO of CAPRIN1 (sgCAPRIN1) increased both APP and Aβ42 levels (Figure 1D,E), supporting CAPRIN1's role in regulating APP degradation and Aβ42 production.

Using the APP695‐HiBiT clones, we screened our CAPRIN1‐targeted compound library, which comprises approximately 219 compounds generated through structure‐activity relationship optimization of derivatives of the CAPRIN1‐targeted lead compound HB007 [29]. Treatment with individual compounds for 24 h reveal several compounds that reduced APP695‐HiBiT levels, with compound 0043 and 0047 being more effective (Figure 1F and Figure S1D). After 72‐h treatment, ELISA and cell viability assays indicated that compound 0043 was the most potent in reducing Aβ42 without inducing cytotoxicity (Figure 1G,H and Figure S1E). These active compounds shared common pharmacophores: a bicyclic ring, a urea core, and a phenyl ring. The urea core was conserved in all the compounds. Regarding the bicyclic ring, the 6‐5 ring system was most favorable for activity, with a benzothiazole ring as found in compound 0043, showing the higher potency (Figure S1F).

To assess target engagement, HEK‐APP695 cells were treated with compound 0043; and APP and CAPRIN1 were isolated by IP using their respective antibodies. Western blot and ELISA showed that 0043 treatment enhanced the interaction between CAPRIN1 and APP (Figure 1I,J) and reduced intracellular APP and extracellular Aβ42 levels in a dose‐dependent manner (Figure 1K,L); whereas these effects were abolished in *CAPRIN1* KO cells (Figure 1M,N). Notably, 0043 treatment had no effect on CAPRIN1 and its binding partner, Ras GTPase‐activating protein‐binding protein 1 (G3BP1) levels [32], and qRT‐PCR revealed no significant changes in APP mRNA (Figure S1G,H), indicating that the compound has no effects on APP mRNA, CAPRIN1 protein levels, and functional activity. In contrast to the CAPRIN1‐targeted SUMO1 degrader HB007 that inhibits cancer cell growth through degradation of SUMO1 [29], 0043 treatment had no effects on cancer cell growth and SUMO1 protein levels in cancer cells (Figure S1I–K). Collectively, the results suggest that the hit compound 0043 enhances the interaction of CAPRIN1 and APP and promotes APP degradation Aβ peptide reduction.

### 2.2 The Hit Compound 0043 Degrades APP and Reduces Aβ Release in AD‐Derived iPSC‐Ns

**FIGURE 2:**
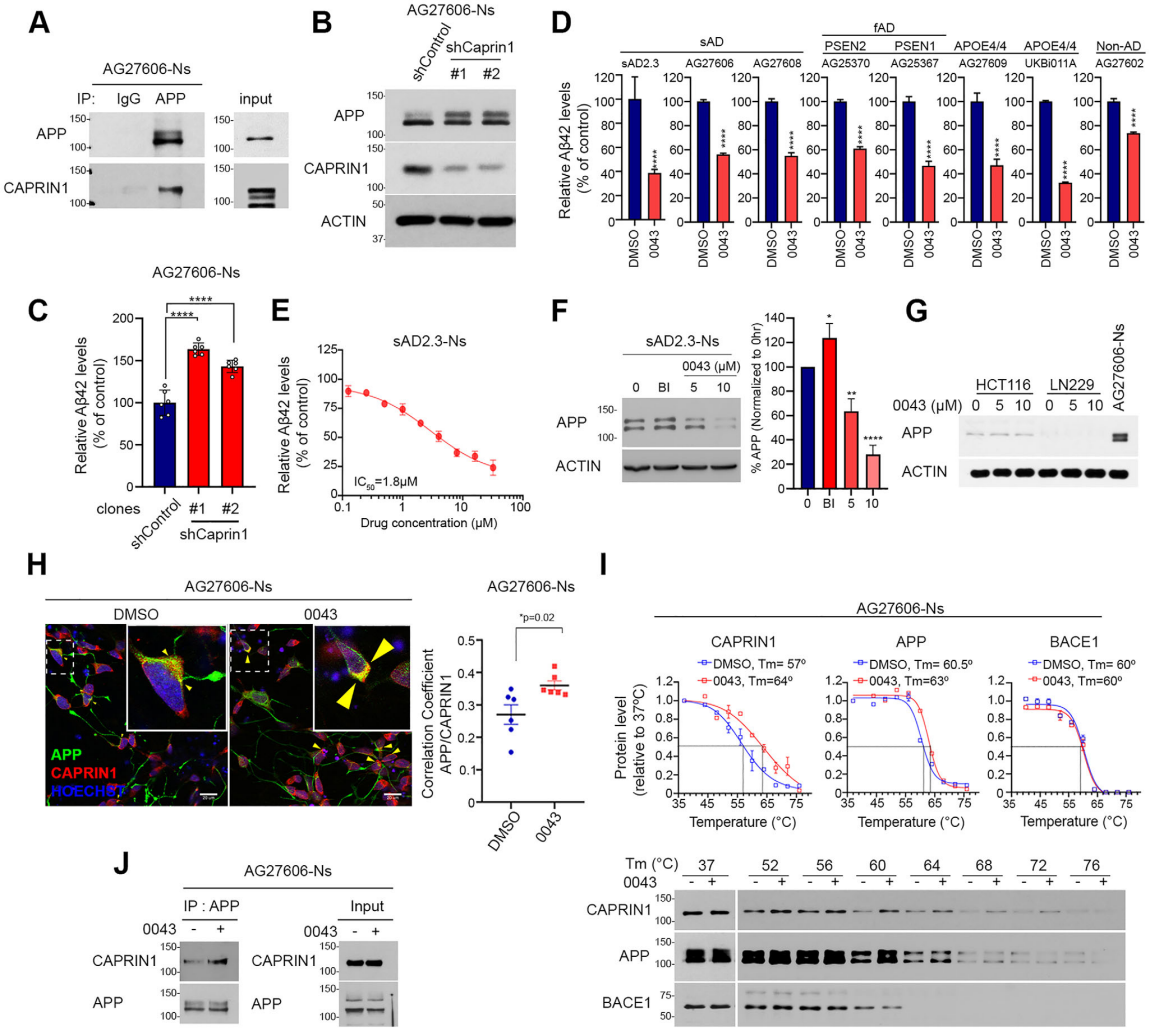
Compound 0043 promotes CAPRIN1‐mediated APP degradation and reduces Aβ42 in AD iPSC‐derived neurons. (A) Endogenous interaction between APP and CAPRIN1 in AG27606 neurons detected by IP. (B) Western blot of AG27606 neurons infected with shControl or shCAPRIN1 lentivirus for 72 h. (C) Relative Aβ42 levels in conditioned media measured by ELISA in culture media from the same cells as in (B) (mean ± s.d.). (D) Relative Aβ42 levels in conditioned media from multiple AD and non‐AD iPSC‐derived neurons treated with DMSO or 0043 (10 µm, 72 h) (mean ± s.d.; *n* = 4; ^****^ *p* < 0.0001; unpaired two‐tailed *t*‐test). (E) Dose–response curve of relative Aβ42 levels in conditioned media in sAD2.3 neurons treated with 0043 for 5 days (IC_50_ = 1.8 µm; mean ± s.d.; *n* = 3). (F) Western blot showing APP levels in sAD2.3 neurons treated with 0043 (5 or 10 µm, 72 h); BACE1 inhibitor (BI, 10 µm) used as negative control. (G) APP levels by western blot in HCT116 and LN229 cells treated with 0043 (10 µm, 72 h); AG27606 neurons used as neuronal control. (H) Confocal images of AG27606 neurons treated with DMSO or 0043 (10 µm, 24 h), stained for APP (green), CAPRIN1 (red), and nuclei (Hoechst, blue). Arrowheads denote colocalization (scale bar, 20 µm). Quantification using Pearson's correlation coefficient (*n* = 6; ^***^ *p* < 0.001; unpaired two‐tailed *t*‐test). (I) Cellular thermal shift assay (CETSA) analysis of endogenous APP and CAPRIN1 in AG27606 neurons treated with 0043 (10 µm); thermal stability curves shown with BACE1 as control. (J) Endogenous APP was immunoprecipitated from AG27606 neurons treated with 0043 (10 µm, 24 h), followed by immunoblotting for CAPRIN1. Aβ42 levels in conditioned media were normalized to the DMSO control.

Human induced pluripotent stem cells (iPSCs) have accelerated disease modeling and drug discovery, and the development of patient‐derived iPSC neurons (iPSC‐Ns) has provided a relevant system to study this complex disease [33]. To assess compound activity in AD neurons, we differentiated iPSCs from sporadic AD (sAD), familial AD (fAD), and non‐AD individuals into mutual neurons using doxycycline‐inducible Neurogenic 2 (Ngn2) and Ascl1 (Msch1) expression (Figure S2A,B) [34, 35]. iPSC‐Ns exhibited neuronal morphology, downregulation of stemness marker NANOG, and upregulation of neuronal marker MAP2 (Figure S2C,D). To examine CAPRIN1's role in AD neurons, we immunoprecipitated APP from sAD‐iPSC‐Ns (AG27606‐Ns) and confirmed its interaction with CAPRIN1 (Figure 2A). No interactions were observed between CAPRIN1 and APP homologues: amyloid precursor‐like proteins 1 and 2 (APLP1 and APLP2) (Figure S2E). *CAPRIN1* knockdown via its shRNAs increased intraneuronal APP and secreted Aβ42 (Figure 2B,C), supporting the role of CAPRIN1 in APP degradation in human AD neurons.

We next evaluated compound activity and showed that both 0043 and 0047 reduced Aβ42 levels, but 0047 was cytotoxic in sAD‐iPSC‐Ns (SAD2.3‐Ns) (Figure S2F,G). Compound 0043 effectively lowered Aβ42 secretion across iPSC‐Ns derived from sAD, fAD and non‐AD iPSCs (Figure 2D). Its activity was dose‐ and time‐dependent with IC_50_ values of 1.8–2.1 µm in SAD2.3‐Ns and AG27606‐Ns (Figure 2E and Figure S2H,I). Western blotting confirmed that 0043 reduced intracellular APP, whereas the BACE1 inhibitor (BI, β‐secretase inhibitor IV) did not in SAD2.3‐Ns and AG27606‐Ns and instead resulted in a modest increase in APP accumulation in SAD2.3‐Ns and AG27606‐Ns (Figure 2F and Figure S2J). Importantly, 0043 did not affect levels of other AD‐related proteins, including BACE1, ADAM10, PSEN1, nicastrin, ADP ribosylation factor‐binding protein 1 (GGA1), Sorla, and neprilysin in SAD2.3‐Ns, nor did it alter APP mRNA expression in AD iPSC‐Ns (Figure S2K,L).

Compound 0043 degraded APP in iPSC‐Ns but not in the glioblastoma cell line LN229 and colorectal cancer cell line HCT116 that lack APP expression (Figure 2G), indicating its neuronal‐specific activity and distinguish it from the CAPRIN‐targeted SUMO1 degrader HB007 [29]. To further assess the mechanism of action, fluorescence confocal microscopy showed increased co‐localization of APP and CAPRIN1 after 0043 treatment in AG27606‐Ns (Figure 2H). Cellular thermal shift assay (CETSA) revealed that 0043 stabilized CAPRIN1 and APP but not BACE1 in both AG27606‐Ns and HEK‐APP695 cells (Figure 2I and Figure S2M). Finally, APP was purified from AG27606‐Ns by IP after 0043 treatment; and western blotting revealed that the compound enhances CAPRIN1‐APP interaction (Figure 2J). Together, these findings demonstrate that compound 0043 binds CAPRIN1 and APP, enhancing their interaction and promoting CARPIN1‐mediated degradation of APP in AD neurons.

### 2.3 SAR Studies of 0043 Derivatives Identify Soluble and Potent Early Lead Compound 0152

**FIGURE 3:**
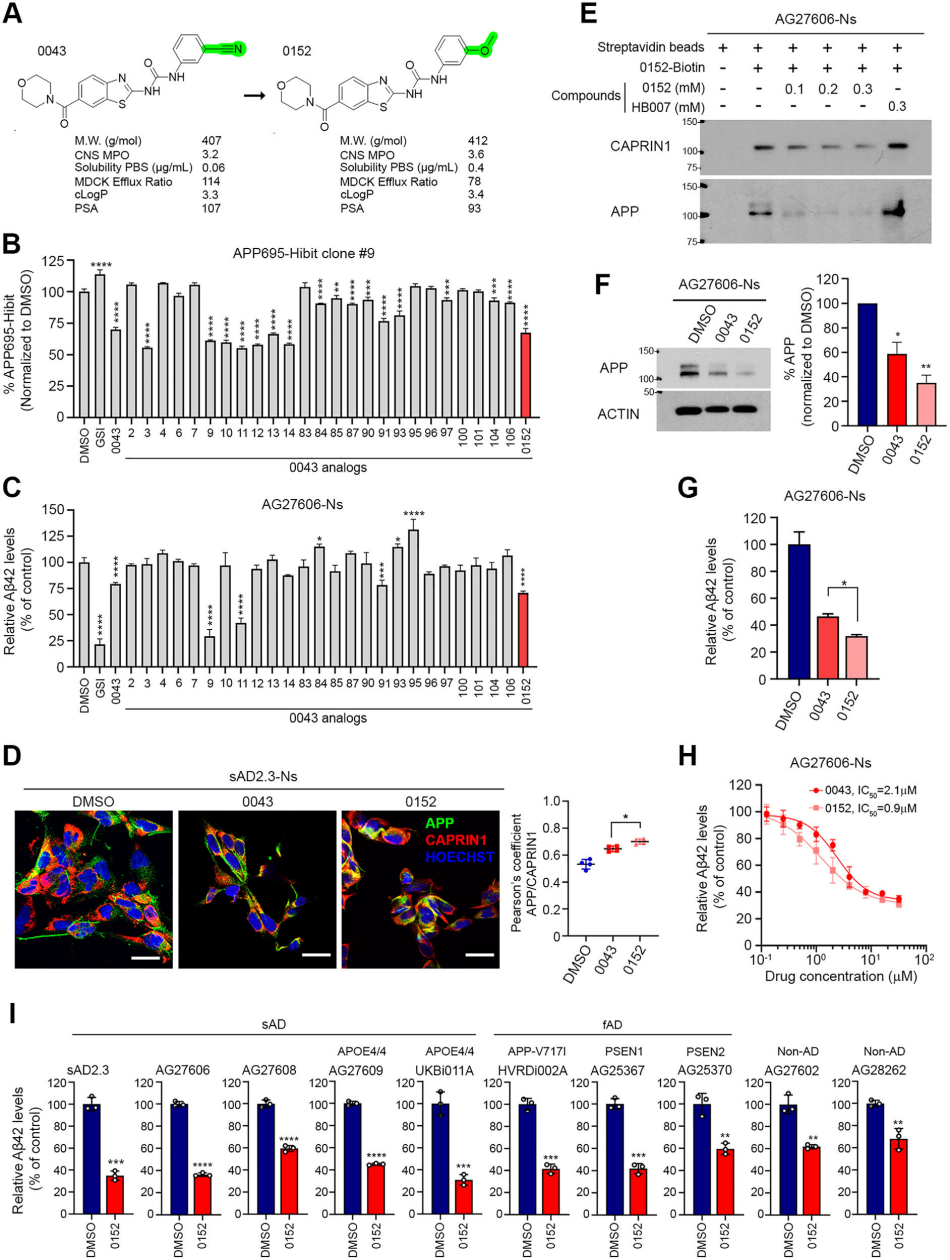
Compound 0152 is a more potent and soluble APP degrader than 0043. (A) Structural comparison of 0043 and 0152, highlighting modifications and physicochemical properties (MW, CNS MPO, PBS solubility, MDCK efflux ratio, cLogP, PSA). MDR1‐MDCKII permeability assays were performed in duplicate (n = 2) in both directions. (B) APP695‐HiBiT signal in HEK293 cells expressing APP695 (HEK‐APP695^WT^) treated with 10 µm 0043 analogs (24 h), shown as % change from DMSO control; 0152 highlighted in red (^****^ *p* < 0.0001). (C) Relative amyloid‐β42 (Aβ42) levels in conditioned media from AG27606 neurons treated with 0043 analogs (72 h), shown as % change from DMSO control; 0152 highlighted in red (^****^ *p* < 0.0001. (D) Confocal images and Pearson's colocalization coefficient for APP and CAPRIN1 in sAD2.3 neurons treated with DMSO, 0043, or 0152 (10 µm, 24 h) (mean ± s.d.; *n* = 4; ^*^ *p* < 0.05). (E) Streptavidin pull‐down assay using biotinylated 0152 in AG27606 lysates. Competitive inhibition with unlabeled 0152 but not control compound HB007. (F) Western blot showing APP reduction in AG27606 neurons after 5‐day treatment with 0152 (10 µm). (G) Aβ42 levels in AG27606 neurons treated with DMSO, 0043, or 0152 (10 µm, 72 h) (mean ± s.d.; *n* = 3; ^*^ *p* < 0.05). (H) Dose–response curves in AG27606 neurons after 5‐day treatment with 0043 or 0152; 0152 showed improved potency (IC_50_ = 0.9 µm vs. 2.1 µm; mean ± s.d.; *n* = 3). All values are normalized to the DMSO‐only control (0.1% DMSO), corresponding to the 0 µm condition. (I) Aβ42 levels in culture media of iPSC‐derived neurons from sAD, fAD (PSEN1/2, V717I), APOE4/4 carriers, and non‐AD controls treated with 0152 (10 µm, 72 h) by ELISA (mean ± s.d., n = 3, ^**^ *p* < 0.01, ^***^ *p* < 0.001, ^****^ *p* < 0.0001).

Compound 0043, which consists of a benzothiazole ring with morphine amide and a phenyl ring bearing a nitrile group, exhibited favorable drug‐like properties in terms of molecular weight (MW), calculated partitional coefficient (cLogP), and polar surface area (PSA) but showed poor aqueous solubility by LC‐MS analysis (Figure 3A). To improve the solubility, we conducted initial structure‐activity relationship (SAR) studies, introducing polar or flexible group and methyl substitutions of the urea linker to disrupt planarity (Figure S3A). A series of derivatives were designed, synthesized, and screened in HEK‐APP695‐HiBiT cells and AG27606‐Ns using luminescence, ELISA, and cell viability assay. To enable a robust and scalable screening workflow, initial compound activity was assessed in HEK‐APP695‐HiBiT cells for rapid quantification of intracellular APP levels, followed by validation in disease‐relevant AG27606‐Ns measuring secreted Aβ42 and toxicity. Among the derivatives, compound 0152 emerged as a more potent and less cytotoxic analog, effectively reducing intracellular APP and extracellular Aβ42 without neuronal toxicity (Figure 3B,C and Figure S3B). To further characterize its activity, compound 0152 showed a dose‐ and time‐dependent reduction in APP695‐HiBiT levels, indicating a robust decrease in APP (Figure S3C). Compound 0152 was modified from compound 0043 by replacing the nitrile with a methoxy group on the phenyl ring. It retained favorable MW, cLogP, and PSA, while significantly improved aqueous solubility (Figure 3A). Central nervous system (CNS) multiparameter optimization (MPO) assessment [36] suggested that compound 0152 had improved blood‐brain barrier (BBB) permeability as compared to 0043. The MDR‐MDCK assay showed that compound 0152 exhibited a lower efflux ratio than 0043, calculated as the ratio of the permeability coefficient in the apical to basolateral direction through the monolayers (Papp, A‐B) (Figure 3A), predicting improved oral absorption and brain penetration of compound 0152 [37].

To examine compound binding to cellular CAPRIN1 and APP, we observed increased colocalization of APP and CAPRIN1 following treatment with either compound 0043 or 0152, with compound 0152 showing greater potency in AG27606‐Ns and sAD2.3‐Ns (Figure 3D and Figure S3D). To assess direct target engagement, compound 0152 was chemically conjugated to biotin, and biotin‐conjugated 0152 (0152‐biotin) was used in pull‐down assays with AG27606‐N lysates. Western blotting confirmed binding of 0152 to both CAPRIN1 and APP, which was competitively inhibited by excess free 0152 but not by the inactive analog HB007 (Figure 3E). Functional assays showed that compound 0152 was more potent than 0043 in reducing both intracellular APP and extracellular Aβ42 (Figure 3F–H and Figure S3E–G). Across a panel of iPSC‐Ns derived from sAD, fAD, and non‐AD individuals, 0152 consistently reduced both APP and Aβ42 levels (Figure 3I and Figure S3H). Together, these findings identify compound 0152 as a promising APP molecular glue degrader with enhanced potency, solubility and BBB permeability. To determine whether these effects are associated with global changes in SUMOylation, we examined SUMO1 levels in neuronal cells and found no appreciable change upon treatment (Figure S3I), suggesting a more target‐specific mechanism of action.

### 2.4 The Compounds Act as Molecular Glues That Enhance CAPRIN1‐APP Interaction

To confirm direct protein‐protein and compound interaction, His‐tagged CAPRIN1 and MBP‐tagged APP695 were purified and sedimented on glycerol gradients (Figure S4A). SDS‐PAGE showed a shift of peak fractions toward higher molecular weights when rhCAPRIN1 and rhAPP were mixed (Figure S4B). IP of the peak fractions by CAPRIN1 antibodies, followed by western blotting with MBP antibody, confirmed a direct interaction of CAPRIN1 and APP (Figure S4C). A ThermoFluor assay showed thermal shifts for both proteins upon 0043 treatment, indicating compound binding of CAPRIN1 and APP (Figure S4D,E). Furthermore, compound 0043 enhanced the interaction between CAPRIN1 and APP, as demonstrated by concentration‐dependent increases in reciprocal co‐immunoprecipitation using specific antibodies (Figure S4F,G).

**FIGURE 4:**
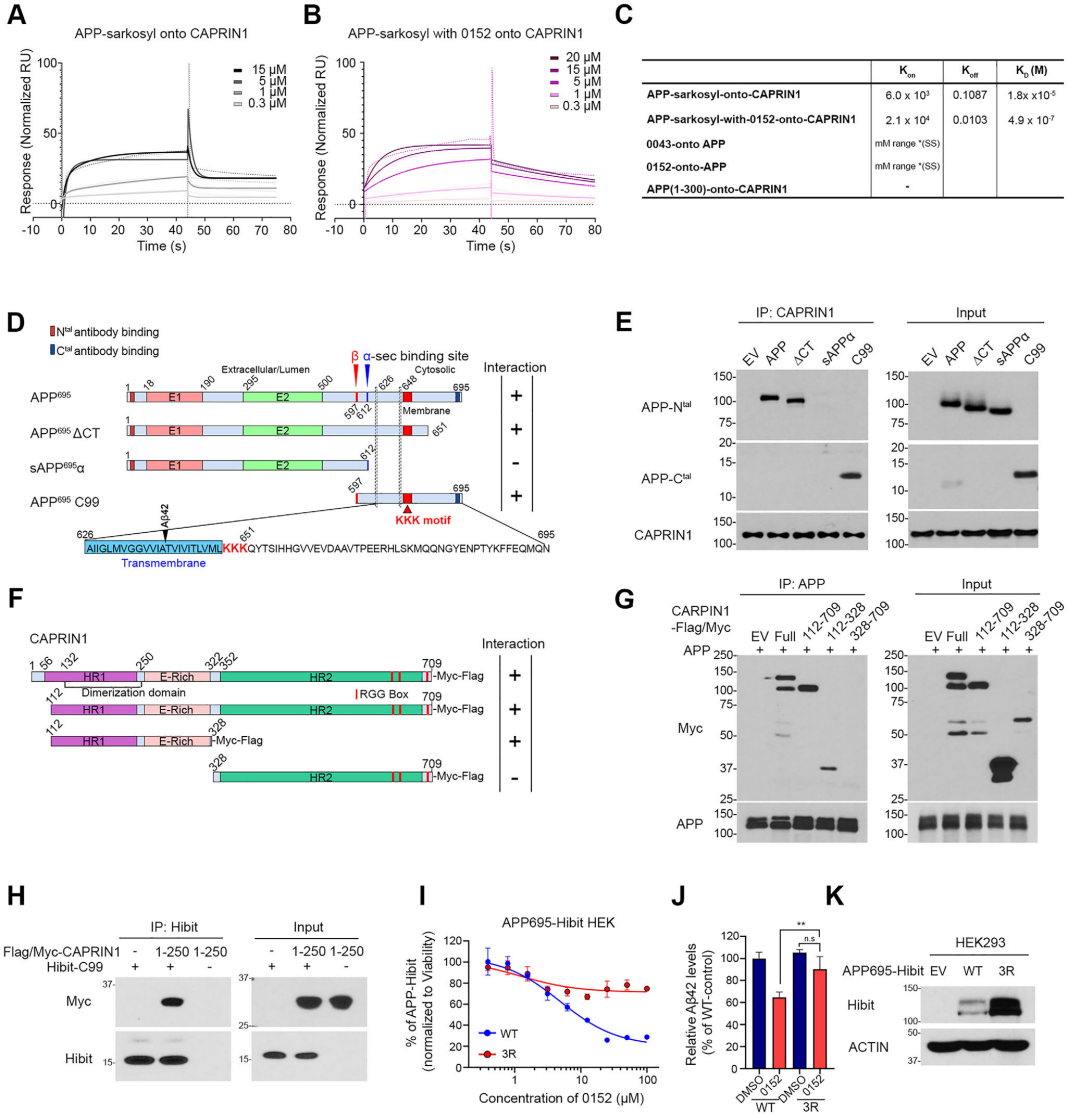
Compound 0152 stabilizes the APP–CAPRIN1 interface in vitro. (A, B) Surface plasmon resonance (SPR) sensorgrams showing binding of sarkosyl‑solubilized APP to immobilized CAPRIN1 in the absence (A) or presence (B) of 0152 (0.1 µm). Experimental data (dotted lines) and fits (solid lines) are shown for increasing APP concentrations. (C) Kinetic parameters (K_D_, equilibrium dissociation constant, M; Kon, association rate constant (M^−^^1^s^−^^1^); Koff, dissociation rate constant (s^−^^1^)) for APP–CAPRIN1 interactions with or without 0152; steady‑state model used for 0043 and 0152 data. (D) Schematic of APP fragments used for CAPRIN1 interaction mapping, indicating cleavage sites, transmembrane domain, and intracellular region. (E) In HEK293 cells expressing APP fragments, CAPRIN1 was immunoprecipitated, and APP fragments were detected by immunoblot using N‐ or C‐terminal APP antibodies. (F) Diagram of CAPRIN1 truncation constructs (full‑length, homology region 1 (HR1), homology region 2 (HR2), glutamate‐rich (E‐rich), and arginine–glycine–glycine (RGG) domains). (G) In HEK293 cells expressing Flag/Myc‐tagged CAPRIN1 truncation constructs, APP was immunoprecipitated, and CAPRIN1 was detected by immunoblot. (H) In HEK293 cells expressing HiBiT‐tagged C99 (HiBiT‐C99) and Flag/Myc‐tagged CAPRIN1 constructs, HiBiT‐tagged C99 was immunoprecipitated, and CAPRIN1 was detected by immunoblot. (I) Dose–response HiBiT assay in APP695‑HiBiT wild‑type (WT) and 3R mutant cells after 72 h treatment (mean ± s.d.; n = 3). (J) Relative amyloid‐β42 (Aβ42) levels in conditioned media of WT and 3R cells treated with DMSO or 0152 (10 µm for 72 hr; mean ± s.d.; n = 3; ^**^ *p* < 0.01; one‑way ANOVA with Tukey's post hoc test). (K) Western blot showing reduced APP degradation in 3R mutant vs. WT cells. Aβ42 levels were normalized to the DMSO control.

To quantify how the compounds enhance the CAPRIN1‐APP interaction, we performed surface plasmon resonance (SPR) using recombinant proteins [38]. rhCAPRIN1 was immobilized on a CM5 sensor chip, and rhAPP695 was applied either before or after incubation with the early lead compound 0152. In the absence of the compound, SPR revealed a concentration‐dependent interaction between APP695 and CAPRIN1. In the presence of equimolar 0152, the dissociation rate constant (K_off_) decreased 10‐fold, while the association rate constant (K_on_) increased 3.5‐fold, leading to a 36‐fold reduction in the equilibrium dissociation constant (K_D_) (Figure 4A–C). These kinetic changes indicate that compound 0152 stabilizes the CAPRIN1‐APP interaction, consistent with a molecular glue mechanism.

To map the interacting regions, we first generated various domain constructs of APP. APP is a membrane protein composed of the E1 domain for adhesion, the E2 domain for dimerization, the Aβ‐secretase cleavage site, the transmembrane domain (TMD), and the intracellular domain including the KKK motif (K649–K651) for cytoplasmic signaling [39]. Each of the constructs was expressed in HEK cells and immunoprecipitated using CAPRIN1 antibodies. Western blotting of the IPs showed that CAPRIN1 interacts with the cytoplasmic domain, including the KKK motif of APP (Figure 4D,E). CAPRIN1 contains two conserved homolog domains (HR1 and HR2), an E‐rich region, Arg‐Gly‐Gly rich (RGG) boxes, and three short RG‐rich repeats [40]. Flag/Myc tagged constructs of CAPRIN1 domains were expressed, together with a full‐length APP in HEK cells. IP with APP antibodies followed by western blotting demonstrated interaction of APP with the N‐terminal HR1 and E‐rich region (Figure 4F,G).

To validate this interaction, we co‐expressed the C‐terminal domain of APP (HiBiT‐tagged C99) and the N‐terminus of FLAG‐tagged CAPRIN1 (residues 1–250) in HEK cells. Flag IP followed by western blotting confirmed the interaction between CAPRIN1 and APP (Figure 4H). To further delineate the interaction interface, we focused on a lysine‐rich (KKK) motif (K649–K651) within APP as a potential CAPRIN1‐interacting region, given its location within the cytosolic domain of C99 and its enrichment in basic residues that may mediate protein–protein interactions [41]. To assess the functional relevance of this interaction in compound activity, we generated an APP695‐3R mutant, replacing the KKK motif (K649‐K651) with arginines by site‐directed mutagenesis. HEK cell stably expressing 3R‐APP695‐HiBiT showed no response to compound 0152, as measured by HiBiT luminescence and Aβ42 ELISA (Figure 4I,J). Western blotting of HEK cells transfected with equal amounts of wild‐type (WT) or 3R‐APP695‐HiBiT DNA revealed higher steady‐state levels of the 3R mutant as compared to WT APP, further supporting the critical role of the KKK motif in CAPRIN1‐mediated APP degradation (Figure 4K). These results demonstrate that compound 0152 functions as a molecular glue by enhancing CAPRIN1–APP association via the KKK motif, which is required for efficient APP turnover. To further assess the role of this motif in APP‐CAPRIN1 interaction, we performed co‐immunoprecipitation experiments comparing WT‐ and 3R‐APP695‐HiBiT. The 3R mutant showed reduced binding to CAPRIN1 relative to WT‐APP (Figure S4H), indicating that this region contributes to APP‐CAPRIN1 interaction, independent of differences in protein stability.

### 2.5 APP Degraders Induce CAPRIN1‐Mediated Lysosomal Degradation of APP in AD iPSC‐Ns

APP is synthesized at endoplasmic reticulum (ER)‐associated ribosomes and trafficked through the secretory pathway to the plasma membrane, where it undergoes proteolytic cleavage to release Aβ peptides [42]. While APP is transported to the cell surface, its amyloidogenic processing occurs predominantly within intracellular compartments, particularly in endosomes where BACE1 and γ‐secretase are active, with additional contributions from Golgi‐associated trafficking pathways. Once at the membrane, APP is internalized into early endosomes (EE), where it can either recycle via cycling endosomes or retrograde transport to the trans‐Golgi network (TGN) or progress to late endosomes (LE) and lysosomes for degradation [43, 44, 45, 46] (Figure S5A). To determine how CAPRIN1 regulates APP degradation, we tracked its intracellular localization using fluorescent confocal microscopy with organelle‐specific markers [47, 48]. In AG27606‐Ns, APP colocalized with the EE antigen 1 (EEA1), LE marker Rab7, and the lysosomal associate membrane protein 1 (LAMP1), and treatment with compound 0043 significantly increased APP colocalization with all three markers (Figure S5B–D), suggesting that the compound enhanced APP trafficking through the endosomal‐lysosomal pathway. CAPRIN1 was colocalized with Rab7 and LAMP1 prior to treatment, and the treatment with compound 0043 increase its colocalization with both markers (Figure S5E–G), suggesting that the compound enhances redistribution of CAPRIN1, like APP, toward the endosomal‐lysosomal pathway.

**FIGURE 5:**
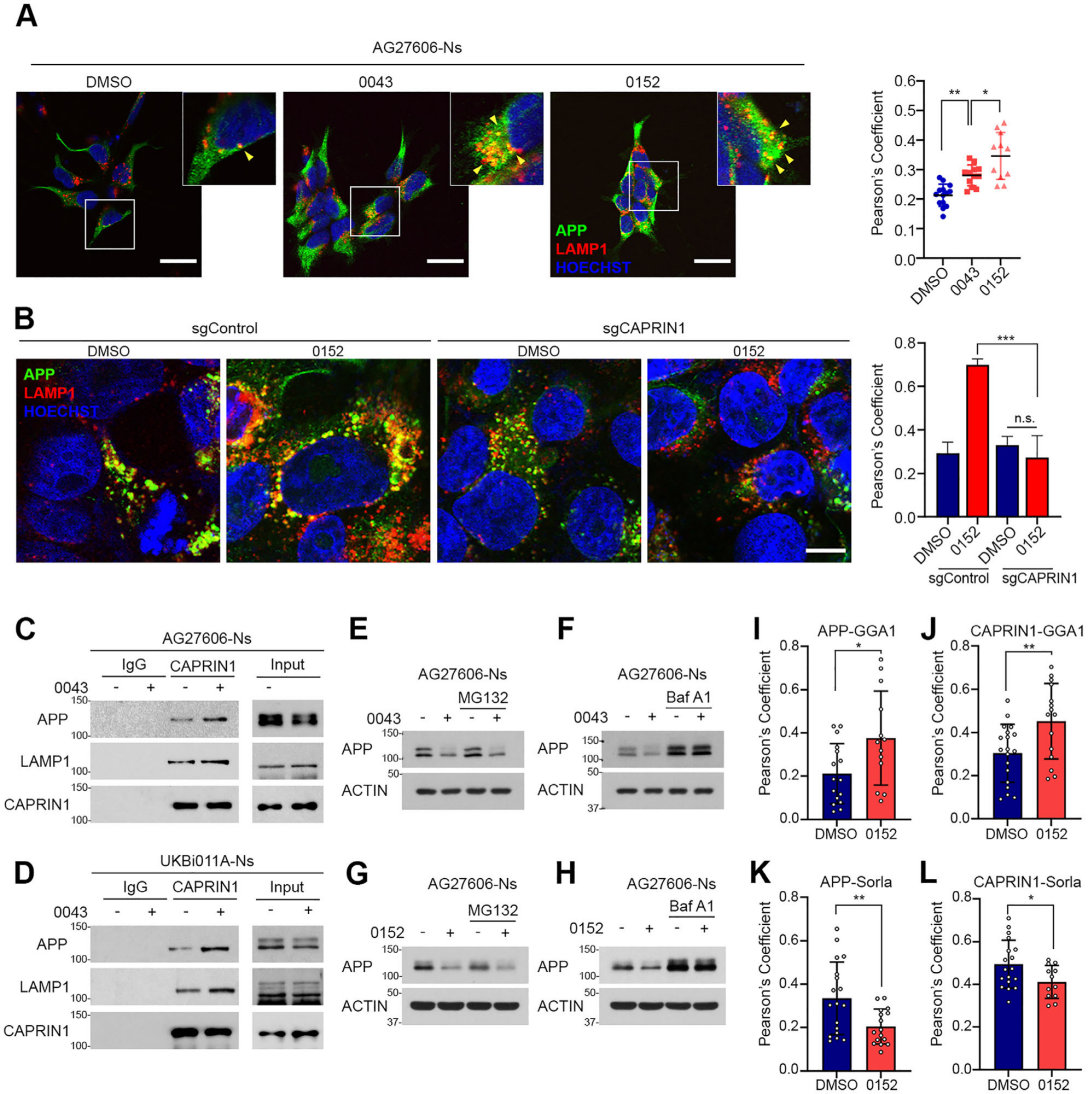
Small‐molecule glue degraders direct APP to lysosomes via CAPRIN1. (A) Confocal images of AG27606 neurons treated with DMSO, 0043, or 0152 (10 µm, 24 h), stained for APP (green), LAMP1 (red) and nuclei (Hoechst, blue). Yellow arrows indicate colocalization (scale bar, 20 µm). Right: quantification of APP–LAMP1 colocalization (mean ± s.d.; ^*^ *p*< 0.05, ^**^ *p* < 0.01). (B) Confocal images of HEK‐APP695^WT^ sgControl or sgCAPRIN1 cells treated with DMSO or 0152, stained for APP and LAMP1 (scale bar, 10 µm). Graph shows Pearson's coefficient of APP–LAMP1 colocalization (mean ± s.d.; ^**^ *p* < 0.001). (C, D) CAPRIN1 was immunoprecipitated from AG27606 (C) and UKBi011A (D) neurons treated with 0043 (10 µm, 24 hr), and co‐precipitated APP and LAMP1 were detected by immunoblot. (E–H) Western blot of APP in AG27606 neurons pretreated with MG132 (5 µm, 30 min) or Bafilomycin A1 (lysosomal inhibitor; 20 nm, 30 min), followed by 0043 or 0152 (10 µm, 24 h). (I, J) Pearson's correlation of APP–GGA1 (I) and CAPRIN1–GGA1 (J) in AG27606 neurons treated with DMSO or 0152 (10 µm, 24 h, mean ± s.d.; ^*^ *p* < 0.05, ^**^ *p* < 0.01; unpaired *t*‐test). (K, L) Pearson's correlation of APP–SORLA (K) and CAPRIN1–SORLA (L) after DMSO or 0152 treatment (10 µm, 24 h, mean ± s.d.; ^*^ *p* < 0.05, ^**^ *p* < 0.01).

Fluorescent confocal microscopy combined with coefficient analysis showed that both compounds 0043 and 0152 increased APP–LAMP1 colocalization, with 0152 showing greater potency across a panel of AD iPSC‐Ns (Figure 5A and Figure S5H,I). Importantly, CRISPR‐mediated KO of CAPRIN1 abolished this compound‐induced colocalization, confirming CAPRIN1's role in mediating APP trafficking to lysosomes (Figure 5B and Figure S5J). IP of CAPRIN1 following 0043 treatment showed increased co‐immunoprecipitation of APP and LAMP1, suggesting their co‐occurrence within a shared trafficking compartment rather than a direct CAPRIN1‐LAMP1 interaction (Figure 5C,D). To further establish APP lysosomal degradation, we pretreated iPSC‐Ns with the lysosome inhibitor Bafilomycin A1 (Baf A1) or the proteasome inhibitor MG132. MG132 had no effect, whereas Baf A1 abolished the APP‐degrading activity of compounds 0043 and 0152 in AG27606‐Ns, sAD2.3‐Ns, and HEK‐APP695 cells (Figure 5E–H and Figure S5K,L). These findings indicate that the compounds promote APP degradation through the lysosomal pathway.

We next examined whether compounds affect APP recycling via the recycling endosome and TGN. Golgi‐associated, gamma‐adaptin ear‐containing, ARF‐binding protein 1 (GGA1) mediates sorting from endosomes to lysosomes [49, 50], while sorting‐related receptor with A‐type repeats (SorLA) promotes recycling from endosomes back to the TGN [49, 51]. Fluorescent confocal microscopy revealed that 0152 treatment enhanced the colocalization of both APP and CAPRIN1 with GGA1, further supporting CAPRIN1‐mediated trafficking of APP from endosomes toward lysosomes (Figure 5I,J and Figure S5M,N). In contrast, 0152 reduced the colocalization of both APP and CAPRIN1 with SorLA (Figure 5K,L and Figure S5O,P). Together with the increased CAPRIN1 colocalization with Rab7 and LAMP1 (Figure S5E–G), these findings indicate that compound 0152 promotes redistribution of CAPRIN1 toward the endosomal–lysosomal pathway in parallel with APP trafficking (Figure S5A).

### 2.6 CAPRIN1–APP Interaction in AD Brains and Organoids With 0152‐Induced APP/Aβ42 Reduction

**FIGURE 6:**
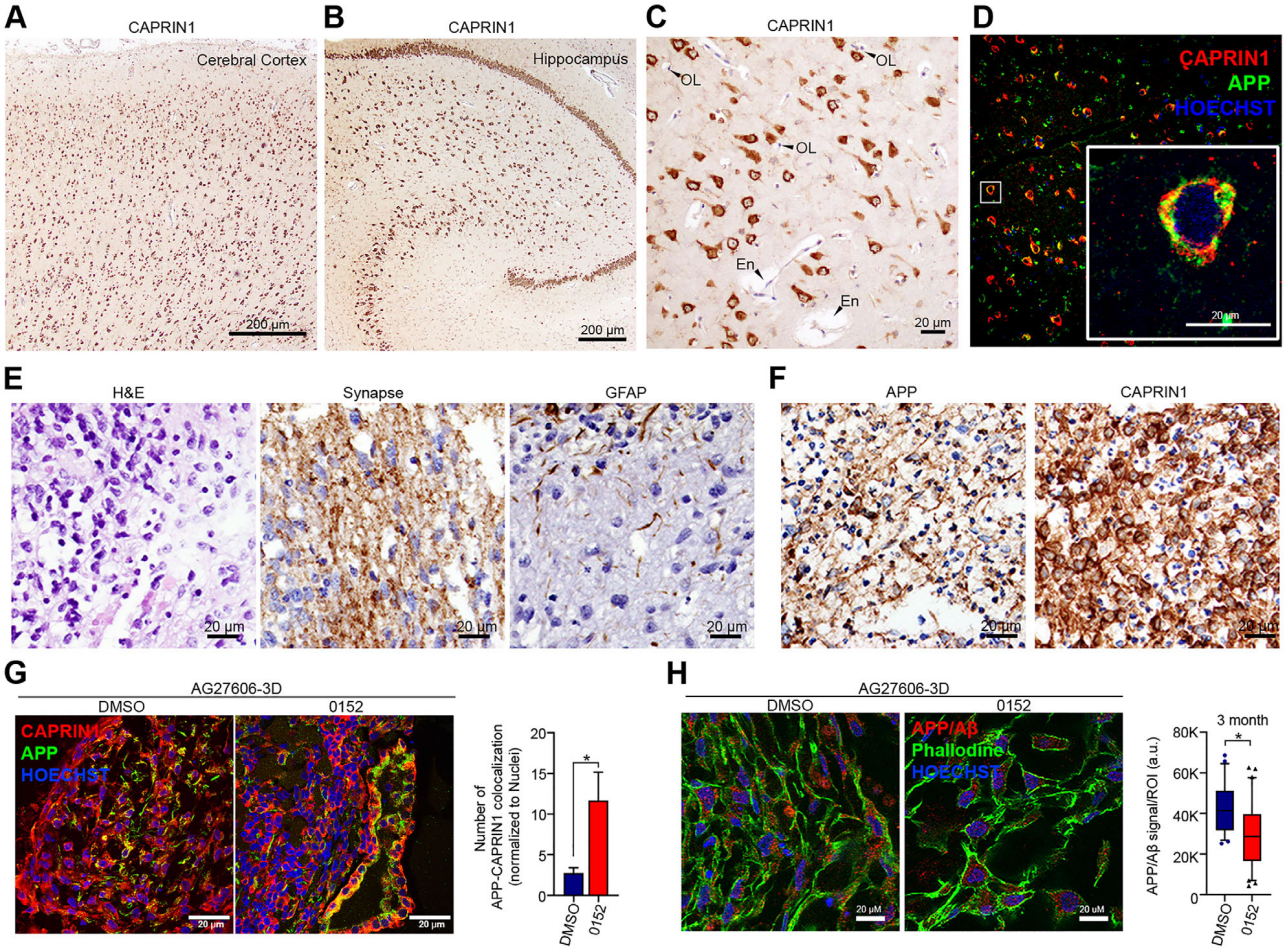
CAPRIN1 and APP co‐localize in AD brain and organoids; 0152 reduces APP/Aβ levels in 3D cortical models. (A, B) Immunohistochemistry (IHC) of CAPRIN1 in postmortem AD brain cortex (A) and hippocampus (B), showing cytoplasmic localization (scale bars, 200 µm). (C) CAPRIN1 in oligodendrocytes (OL) and endothelial cells (En) in AD cortex (scale bar, 20 µm). (D) Confocal immunofluorescence of APP (green) and CAPRIN1 (red) with Hoechst (blue) in human AD brain (scale bar, 20 µm). (E) Hematoxylin and eosin (H&E) and IHC for synaptophysin and glial fibrillary acidic protein (GFAP) in AG27606 cortical organoids (scale bar, 20 µm). (F) IHC of APP and CAPRIN1 in 12‐week‐old organoids (scale bar, 20 µm). (G) Confocal images of 3‐month AG27606 organoids treated with DMSO or 0152 for 3 months; stained for APP (green), CAPRIN1 (red), Hoechst (blue) (scale bar, 20 µm). Right: quantification of APP–CAPRIN1 colocalization (mean ± s.d.; ^*^ *p* < 0.05). (H) Confocal images of organoids stained for APP/Aβ (4G8; red), phalloidin (green) and Hoechst (blue) after DMSO or 0152; quantification of APP/Aβ immunoreactivity (mean ± s.d.; ^*^ *p* < 0.05).

To assess the role of CAPRIN1 in AD, we analyzed the AMP‐AD (Accelerating Medicines Partnership Alzheimer's Disease)/AGORA human brain proteomic dataset to determine whether APP and CAPRIN1 expression levels are altered in human AD brains as compared with no‐AD brains. APP protein abundance was significantly increased (log2FC = 0.657, adjusted *p* = 2.98 × 10^−16^), whereas CAPRIN1 protein abundance was largely preserved, with only a minimal reduction (log2FC = ‐0.042, adjusted *p* = 5.88 × 10^−3^ (Figure S6A). We next examined the expression and localization of CAPRIN1 in postmortem brain tissue. Immunohistochemistry (IHC) of formalin‐fixed, paraffin‐embedded sections showed that CAPRIN1 was predominantly expressed in neurons of the cerebral cortex and hippocampus in AD and non‐AD brains (Figure 6A,B and Figure S6B,C). CAPRIN1 expression was absent in oligodendroglia and vascular endothelial cells, with weak positivity observed in a subset of astrocytes in both cerebral cortex and subcortical white matter (Figure 6C and Figure S6D). CAPRIN1 was also detected in ependymal and choroid plexus epithelial cells (Figure S6D,E). As expected, APP was mainly neuronal, and immunofluorescence confirmed colocalization of CAPRIN1 and APP in neurons from AD brain sections (Figure 6D and Figure S6F).

To model AD brain tissue and assess compound activity, we generated 3D brain organoids from AD patient‐derived iPSCs (AG27606 and UKBi011A) using established protocols [52]. Ngn2/Ascl1‐iPSCs were cultured to form embryoid bodies (EBs) for five days, induced with doxycycline for five days, embedded in Matrigel, and matured for 12 weeks (Figure S6G). Hematoxylin and eosin (H&E) and IHC confirmed neuronal and astrocytic differentiation, marked by the expression of synaptophysin and glial fibrillary acidic protein (GFAP), respectively (Figure 6E). IHC further showed the expression of CAPRIN1 and APP in neurons in the organoids (Figure 6F). After the 12‐week maturation, organoids were treated with compound 0152 for an additional three months. Confocal microscopy and colocalization coefficient analysis of frozen sections showed that 0152 increased CAPRIN1–APP colocalization in organoids (Figure 6G). Furthermore, staining with an antibody recognizing both APP and Aβ42 revealed that 0152 treatment reduced APP/Aβ42 levels in organoids derived from both AG27606 and UKBi011A iPSCs (Figure 6H and Figure S6H). To directly assess Aβ levels independent of immunostaining, we quantified Aβ42 in organoids by ELISA. Compound 0152 significantly reduced Aβ42 levels in both RIPA‐soluble and formic acid–solubilized fractions (Figure S6I). These studies demonstrate that CAPRIN1 and APP are expressed and colocalized in neurons of both AD brains and organoids. Importantly, compound 0152 enhances this protein‐protein interaction and reduces APP/Aβ42 accumulation in AD iPSC‐derived organoids.

### 2.7 Compound 0152 Reduces Aβ and Amyloid Plaques in AD Mouse Brains

**FIGURE 7:**
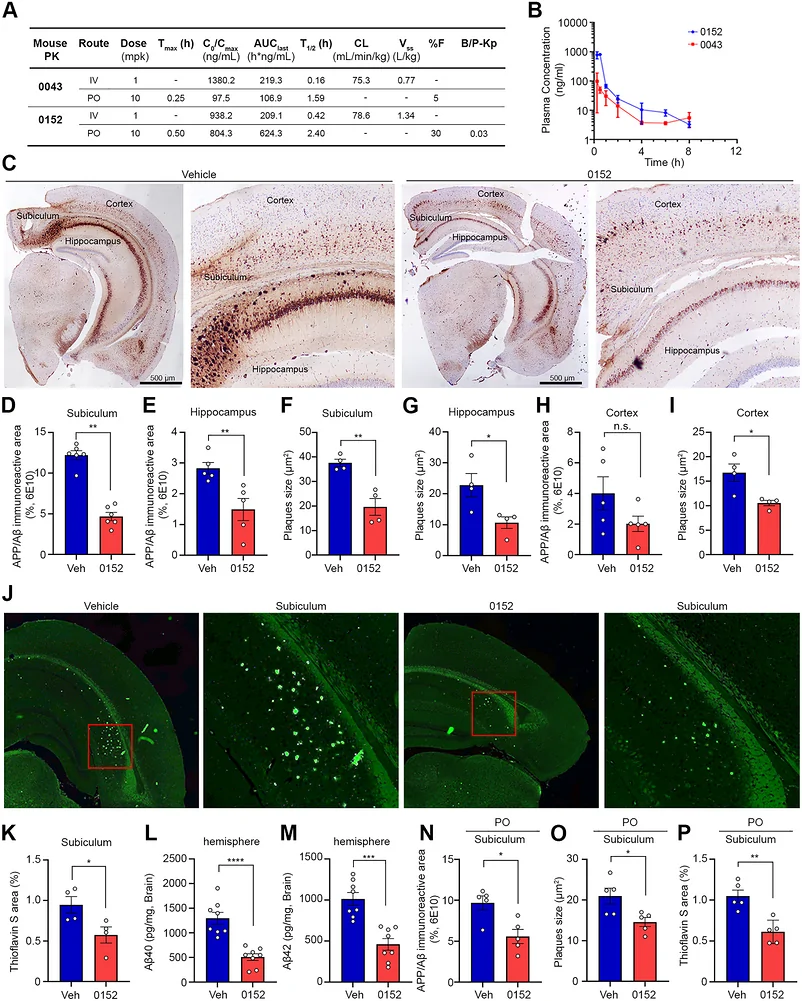
Compound 0152 reduces amyloid burden in 5xFAD mice. (A) Pharmacokinetic parameter of 0043 and 0152 after intravenous (IV) and oral (PO) administration, showing improved bioavailability (%F), half‐life (T_1_/_2_), and clearance (CL) for 0152. (B) Plasma concentration–time profiles following a single PO dose (100 mg/kg) in mice (*n* = 3 per group). (C) Immunohistochemistry (IHC) images of APP/Aβ immunoreactivity in cortex, hippocampus, and subiculum after 24‐day treatment with vehicle or 0152 (100 mg/kg, intraperitoneal (IP)); representative whole‐brain and subiculum sections shown (scale bars, 500 µm). (D, E) Quantification of APP/Aβ‐positive area (%) in subiculum (D) and hippocampus (E) of 5xFAD mice treated with 0152 (100 mg/kg, IP). (F, G) Quantification of individual plaque size in subiculum (F) and hippocampus (G). (H, I) Quantification of APP/Aβ area (H) and plaque size (I) in cortex. (J, K) Thioflavin S (Thio S) staining of brain sections (J) and quantification in subiculum (K) of vehicle‐ and 0152‐treated mice (^*^ *p* < 0.05; unpaired *t*‐test). (L, M) Quantification of Aβ40 (L) and Aβ42 (M) in hemispheric brain lysates from 5xFAD mice treated with vehicle or 0152 (100 mg/kg, IP), measured by ELISA. mean ± s.e.m.; ^***^ *p* < 0.001, ^****^ *p* < 0.0001; unpaired *t*‐test. (N, P) Subicular amyloid pathology in 5xFAD mice (vehicle vs. 0152, 80 mg/kg, PO, 30 days): Quantification of (N) APP/Aβ immunoreactive area detected by the APP (6E10) antibody, (O) plaque size, (P) fibrillar amyloid plaque area measured by Thioflavin S (ThioS) staining. Mean ± s.e.m.; ^*^ *p* < 0.05; unpaired *t*‐test.

To evaluate in vivo activity, we first assessed the pharmacokinetics (PK) of compounds 0043 and 0152. Mice were dosed by oral gavage or intravenous injection, and plasma samples were collected over time for LC‐MS analysis. PK profiling showed that compound 0152 achieved significantly greater drug exposure, with an oral bioavailability (F%) reaching up to 30% compared to only 5% for 0043 (Figure 7A,B), indicating improved drug‐like properties of compound 0152 compared to 0043. In evaluation of BBB permeability, oral administration of 0152 demonstrated its BBB permeability as evidenced by a favorable brain‐to‐plasma (B/P) partition co‐efficient (Kp) [53].

To assess effects on amyloid burden, we used nine‐week‐old 5xFAD mice, corresponding to the early phase of amyloid plaque deposition following initial intraneuronal Aβ accumulation, when Aβ levels increase rapidly due to expression of mutant human APP and PS1 genes [54]. 5xFAD mice were subjected to three different treatment protocols, designed to evaluate efficacy, optimize dosing strategy, and assess the translational potential of this newly designed compound. In the first protocol, 9‐week‐old 5xFAD mice received daily intraperitoneal injections of 100 mg/kg 0152 for 24 days. IHC of brain sections using anti‐APP antibody revealed significant reductions in APP/Aβ‐positive area and amyloid plaque size in the hippocampus and subiculum (Figure 7C–G). The cortex, where amyloid deposition occurs later [54] showed minimal changes in plaque burden (Figure 7H,I). Thioflavin S staining confirmed reduced fibrillar Aβ plaques in the subiculum (Figure 7J,K). ELISA of brain lysates corroborated the IHC results, showing marked decreases in both Aβ40 and Aβ42 levels in the hemispheres (Figure 7L,M). Throughout the treatment period, mice tolerated treatment well, with no observed signs of lethargy, ataxia, paralysis, seizures, or weight loss (Figure S7A).

In the second experiment, 9‐week‐old 5xFAD mice were injected intraperitoneally with 80 mg/kg of 0152 twice per day for 30 days, which was well tolerated with no weight loss (Figure S7B). IHC analysis confirmed that 0152 treatment significantly reduced APP/Aβ‐positive areas and amyloid plaque sizes in the subiculum and hippocampus, as well as amyloid plaque sizes in the cortex (Figure S7C–I). Thioflavin S staining further demonstrated that 0152 treatment drastically reduced fibrillar Aβ plaques in the subiculum (Figure S7J,K). In a third protocol, 9‐week‐old 5xFAD mice were treated via oral gavage with 80 mg/kg of 0152 twice daily for 30 days. IHC and Thioflavin S staining demonstrated a significant reduction of APP/Aβ‐positive areas and plaque sizes in the subiculum of 0152‐treated mice compared to controls, with no observed weight loss (Figure 7N–P and Figure S7L–O). These findings provide strong in vivo evidence that compound 0152 reduces Aβ accumulation and amyloid plaque formation in the 5xFAD AD mouse model.

## 3 Discussion

Over the past decades, therapeutic strategies for AD have primarily focused on inhibiting BACE1 and γ‐secretase to reduce Aβ peptide production. However, clinical trials of BACE1 and γ‐secretase inhibitors have encountered significant setbacks due to limited efficacy and off‐target effects, as these enzymes are involved in physiological processes beyond Aβ generation [55]. More recently, mAbs targeting Aβ have been developed to facilitate amyloid clearance. Clinical trials of Aβ‐directed mAbs, including aducanumab, lecanemab, and donanemab, have demonstrated their ability to reduce amyloid burden and modestly slow cognitive decline in early‐stage AD patients [5, 6, 7]. Nonetheless, these treatments are associated with substantial risks such as cerebral hemorrhage and amyloid‐related imaging abnormalities, highlighting the urgent need for more effective and safer therapeutic strategies [56].

Antisense oligonucleotides (ASOs) have recently emerged as a strategy to modulate *APP* mRNA, resulting in decreased APP expression and reduced Aβ production in iPSC‐derived neurons [57]. The clinical success of ASO therapies in spinal muscular atrophy provides a strong translational precedent [58] and early‐phase trials of intrathecal tau‐targeting ASOs in patients with mild AD have shown encouraging results [59]. Despite these advances, ASO‐based strategies, like Aβ‐targeted mAbs, face a significant limitation in AD: poor penetration of the BBB, which restricts delivery to deep brain tissues [60, 61]. In contrast, we demonstrate that APP‐targeted molecular glue degraders reduce intraneuronal expression of APP and extracellular release of Aβ, and upon oral administration, the lead compound 0152 can cross the BBB and significantly reduce Aβ production and amyloid burden in the brains of 5xFAD mice.

TPD technologies have been predominantly applied to oncoproteins but have recently begun to expand into neurodegenerative diseases [62]. PROTACs have been developed to target tau and were shown to promote CRBN‐mediated degradation of pathological tau in AD mouse models [63] as well as in patient‐derived neuronal cells from frontotemporal dementia [64]. In addition, Von Hippel–Lindau (VHL)‐recruiting PROTACs have been developed to degrade leucine‐rich repeat kinase 2 (LRRK2), a key pathogenic protein implicated in Parkinson's disease [65]. Both PROTACs and autophagy‐targeting chimeras (AUTOTACs) have also been shown to induce degradation of α‐synuclein through ubiquitin–proteasome and autophagy–lysosomal pathways, respectively [66, 67]. LYTACs have further been reported to target Apolipoprotein E4 and direct this membrane‐associated protein to the lysosomal pathway for degradation [68]. In the present study, we identify compounds 0043 and 0152 as the first lysosome‐directed molecular glue degraders that bind at the CAPRIN1–APP interface, enhance the CAPRIN1–APP interaction, and facilitate CAPRIN1‐mediated degradation of APP through the endo‐lysosomal pathway. Other approaches were reported to reduce APP by enhancing lysosomal degradation, including activation of autophagy and stimulation of TFEB‐dependent lysosomal biogenesis [69, 70]. While these strategies broadly enhance lysosomal or autophagic pathways and influence the turnover of multiple cellular substrates, our findings reveal a mechanistically distinct pathway. Rather than globally activating lysosomal function, compound 0152 acts as a molecular glue that enhances CAPRIN1‐APP interaction and promotes CAPRIN1‐mediated trafficking of APP toward lysosomes for degradation. This substrate‐directed mechanism represents a conceptually distinct strategy from global lysosomal activation, providing a targeted approach for APP degradation.

Molecular degraders harness endogenous degradation pathways, primarily the ubiquitin–proteasome system predominantly for cytosolic proteins and the endo‐lysosomal system mainly for membrane‐associated proteins, to selectively reduce pathogenic proteins [71, 72]. Advances in computational ligand‐based drug design have enabled the development of homologous compound series that interface with an E3 ligase and multiple target proteins, effectively expanding the “degradable proteome” or “degrome” [73]. Previously, we reported HB007, a molecular glue degrader that targets CAPRIN1 and FBXO42, which mediates the recruitment of the FBXO42 substrate SUMO1 to the FBXO42‐CUL1 E3 ligase and proteasomal degradation in cancer [29]. In the present study, we discovered a previously unrecognized role of CAPRIN1 in neurons: facilitating lysosomal degradation of APP through direct interaction. Notably, the distinct cellular activities of our SUMO1 and APP degraders likely reflect the cell‐type‐specific expression of their respective targets, in which APP is not expressed in cancer cells, while the FBXO42–CUL1 complex is largely absent in neurons [29].

In this study, we demonstrate that compounds 0043 and 0152 function as lysosome‐directed molecular glue degraders of APP. Classical molecular glue degraders are monovalent small molecules that stabilize interactions between E3 ligases and substrate proteins, thereby promoting ubiquitination and proteasomal degradation. In contrast, compounds 0043 and 0152 neither recruit an E3 ligase nor engage the ubiquitin–proteasome system. Instead, our complementary biochemical, cellular, and functional studies support a distinct molecular‐glue mechanism in which these compounds stabilize the interaction between CAPRIN1 and APP to promote CAPRIN1‐mediated lysosomal degradation of APP. First, we expressed and purified rhCAPRIN1 and rhAPP, respectively (Figure S4A), and demonstrated a direct interaction between rhCAPRIN1 and rhAPP by glycerol‐gradient sedimentation (Figure S4B) and immunoprecipitation (IP) analysis (Figure S4C). The CAPRIN1–APP interaction was further demonstrated in protein‐expressing cellular models (Figure 1I,J) and AD iPSC‐Ns (Figure 2A), while CAPRIN1/APP colocalization was observed in cortical neurons of postmortem AD brains (Figure 6D). These findings establish the biochemical and cellular basis for the formation of the CAPRIN1–APP complex. Next, ThermoFluor analysis demonstrated direct binding of compound 0043 to rhCAPRIN1 and rhAPP, respectively (Figure S4D,E). Consistent with these findings, a biotinylated compound 0152 (biotin‐0152) pulled down endogenous CAPRIN1 and APP from lysates of AD iPSC‐Ns (Figure 3E), demonstrating cellular engagement of both proteins. Importantly, SPR analysis quantitatively demonstrated that compound 0152 stabilizes the interaction between rhCAPRIN1 and rhAPP and promotes formation of the ternary CAPRIN1–0152–APP complex (Figure 4A–C). Thus, compound‐dependent stabilization of the CAPRIN1–APP interaction represents the defining molecular‐glue feature of this system. Furthermore, compounds 0043 and/or 0152 increased the CAPRIN1–APP interaction in protein‐expressing cells and AD iPSC‐Ns (Figures 1J, 2I,J, and Figure S2M). Finally, functional studies throughout this manuscript demonstrate that enhancement of the CAPRIN1–APP interaction by these compounds promotes APP degradation through the endo‐lysosomal pathway. Collectively, the biochemical, cellular, and functional data support a consistent mechanistic sequence: compound binding → stabilization of the CAPRIN1–APP interaction → CAPRIN1‐dependent lysosomal degradation of APP → reduction of amyloid‐β. Together, these findings define compounds 0043 and 0152 as lysosome‐directed, CAPRIN1‐mediated molecular glue degraders of APP and extend the molecular glue paradigm beyond the canonical ubiquitin–proteasome pathway to lysosomal protein degradation. This expanded molecular‐glue strategy may provide a framework for targeting membrane‐associated proteins and broadening the therapeutic potential of targeted protein degradation.

However, several limitations should be acknowledged. The crystal structure of CAPRIN1 is currently available only for its N‐terminal dimerization domain (residues 132–251) [32], whereas structural information for APP is limited to its extracellular E1 and E2 domains [74]. Further structural studies of full‐length CAPRIN1, APP, and their ternary complex with the compounds will be required to define the molecular basis of glue‐mediated target engagement. In addition, although Aβ peptides are generated from APP during AD pathogenesis, APP also plays essential physiological roles in synaptic plasticity and neuroprotection [4], raising important safety considerations for therapeutic APP degradation. Encouragingly, APP‐targeting antisense oligonucleotides (ASOs) have been shown to reduce APP expression to physiologically tolerated levels without apparent deleterious effects on neuronal function [57]. Consistent with this therapeutic window, oral administration of compound 0152 crosses the blood–brain barrier, significantly reduces Aβ production and amyloid burden in the brains of 5xFAD mice, and does not produce overt toxicity. Nevertheless, further lead optimization to improve solubility, brain penetration, and drug‐like properties will be required to enable dose optimization and to determine the extent of APP degradation needed to suppress the Aβ cascade while preserving the physiological functions of APP in AD mouse models.

In the present studies, we uncover a novel functional role for CAPRIN1 in the regulation of APP homeostasis in neurons. APP is synthesized in the endoplasmic reticulum and trafficked to the plasma membrane, where it undergoes sequential cleavage to release Aβ peptides [42]. Following endocytosis, membrane‐bound APP is routed to the endo‐lysosomal system for degradation. We show here that CAPRIN1 is predominantly expressed in cortical and hippocampal neurons of human brains. CAPRIN1 interacts with membrane‐bound APP, and the cytoplasmic KKK motif may contribute to this interaction. CAPRIN1 is associated with APP trafficking from endosomes to lysosomes and may facilitate its degradation. This interaction appears to be selective, as no binding was observed between CAPRIN1 and the APP homologues APLP1 or APLP2. Our compounds bind CAPRIN1 and APP, stabilize their interaction, and enhance the endo‐lysosomal degradation of membrane‐bound APP without affecting cytosolic CAPRIN1 protein, consistent with the multifunctional functions of CAPRIN1 in neurons.

This study provides proof‐of‐concept for a CAPRIN1‐dependent APP degradation and establishes CAPRIN1‐targeted molecular glues as a novel therapeutic strategy for AD. Using our TPD‐based drug discovery platform [29], we developed cell‐based screening assays that identified compound 0043 as the hit compound. Medicinal chemistry optimization yielded compound 0152, which exhibited improved potency, solubility, and BBB permeability, supporting its suitability for in vivo applications. These compounds promoted CAPRIN1‐mediated lysosomal degradation of wild‐type and mutant APP in neurons differentiated from a panel of AD patient iPSCs. In vivo, treatment with compound 0152 significantly reduced Aβ accumulation and amyloid plaque formation in the brains of 5xFAD mice. Ongoing efforts are focused on optimizing the pharmacokinetic and pharmacodynamic profiles of these compounds to further enhance their potency and drug‐like properties for drug development. Collectively, our findings uncover a previously unrecognized CAPRIN1‐mediated lysosomal degradation pathway for APP in human neurons and introduce a first‐in‐class series of APP‐targeted molecular glue degrader compounds. This strategy represents a novel therapeutic avenue for AD, with the potential to overcome limitations associated with existing Aβ‐directed therapies.

## 4 Experimental Section

### 4.1 Stable APP695 HEK293 Cells

HEK293 cells were purchased from the American Type Culture Collection (ATCC). Lentiviral plasmids (pLV‐Hygro‐CMV‐hAPP, pLV‐Hygro‐CMV‐hAPP‐Hibit, and pLV‐Hygro‐CMV‐hAPP ‐3K to 3R‐Hibit) for APP695WT HEK293 and APP695‐Hibit HEK293 were generated in the third‐generation plasmid. HEK293T cells were transfected with the targeting plasmid and third‐generation packaging plasmid (pMD2.G; addgene#12259, pMDLg/pRRE; Addgene#12251, and pRSV‐Rev; Addgene #12253) together using Lipofectamine 2000. HEK293 cells were infected with virus particles and selected with hygromycin B (200 µg/mL, Invitrogen). For single clone selection, the cells were reseeded in 96‐well plates as single cells. Once expanded, clones were analyzed by ELISA for amyloid beta 42 (Aβ42), western blot for APP expression, and Hibit‐lytic assay (Promega). For sgCAPRIN1 HEK293 cell lines, the guide RNA sequences of CAPRIN1 were cloned into the LentiCRISPRv2 plasmid (Addgene #52961) according to protocol to protocol [75] and the sequences for CAPRIN1‐CRISPR (in Table S1). HEK293 cells were infected, selected with puromycin (1 µg/mL) for 2 days, single‐cell selected, and followed by western blot for CAPRIN1 knock‐out verification. Multiple independent clones were screened, and representative clones were used for subsequent experiments.

### 4.2 Human iPSCs and Neuronal Differentiation

The human iPSC SAD2.3 (UCSD234i‐SAD2‐3) and HVRDi002A were purchased from Wicell, UKBi011A from the European Bank for induced pluripotent stem cells, Sigma, AG25367, AG25370, AG27606, AG27608, AG27609, AG28049, AG28262, and AG27602 from Coriell. All iPSCs were maintained and expanded on Matrigel (Growth factor reduced Matrigel matrix, Corning) in mTeSR1 complete medium (mTeSR1 medium + 5X supplement, Stem Cell Technologies). For iPSC‐Ns transgene delivery, each iPSC line was transduced with lentiviral particles for pLVX‐UbC‐rtTA‐Ngn2:2A:Ascl1 (Addgene #127289) #127289) [76, 77], followed by selection in the presence of puromycin (0.5 mg/mL, Gibco). To initiate conversion, iPSCs were differentiated into neurons using the established Ascl1/Mash1 (33) and Neurogenin 2 (Ngn2) (34) protocols. The transgene‐delivered iPSCs were dissociated with StemPro Accutase (Gibco) and reseeded in Neuro culture media (Neurobasal media (Gibco), 2% B27 supplement (Gibco), 1X Glutamax (Gibco)) with doxycycline (2 µg/mL, Sigma–Aldrich) for 5 days and without doxycycline for 3 days [78], and differentiated iNS exhibited differentiated neuronal morphology.

### 4.3 IPSC‐Derived Brain Organoids

For generating brain 3D organoids, Lancaster's protocol [79] and Paşca's protocol [52] were used with minor modifications. In brief, 9000 cells/ 100 µL of each well were seeded in Ultra‐low attachment 96 well U‐bottom plates (Sbio) supplemented with 10 µm of ROCK inhibitor. The next day, 100 µL of mTeSR1 was added, and the cells were cultured as embryonic bodies (EB) for 5 days at 37°C in 5% CO2 incubator. When the size of EB reached 400–600 µm, EBs were transferred into ultra‐low binding 24 well (Corning) with 200 µL of neural differentiation medium (Neurobasal medium (Gibco), 2% B27, 1X Glutamax) with 2 µg/mL of Doxycycline and incubated for 5 days at 37°C in 5% CO2 incubator until the size of EB was 700–800 µm. During this step, one EB was in one well, and the culture medium was changed every day with fresh neural differentiation medium with Doxycycline. At day 10, the EB was embedded into 15 µL of 100% Matrigel (Corning), and the embedded EBs were cultured in neural differentiation medium without Doxycycline in 6 well ultra‐low binding plates (Corning). In this step, each well of 6 well plates had 16 EBs, and plates were incubated in stationary methods for 5 days at 37°C in 5% CO2 incubator to expand neuroepithelial buds, followed by placing the plates on an orbital shaker (Benchmark Scientific) at 75 rpm at 37°C in 5% CO2 incubator. The fresh medium was changed every 3 days.

### 4.4 Animal Experiments

Female 5xFAD transgenic mice (9–10 weeks old) were used to evaluate the efficacy of 0152 in reducing amyloid pathology. Mice were housed under Indiana University standard laboratory conditions. For intraperitoneal (IP) administration, mice received either vehicle or 0152 (indicated concentrations) in a formulation consisting of 20% DMSO, 20% Cremophor EL, and 60% PBS. For oral (PO) administration, mice received either vehicle or 0152 (indicated concentrations) in a formulation consisting of 10% NMP (N‐methyl‐2‐pyrrolidone), 10% PG (Propylene Glycol), 5% Solutol HS‐15, and 75% Captisol (10% w/v in RO water). At the end of the treatment period, mice were sacrificed, and their brains were collected for histological analysis. Brain tissues were fixed in 10% Formalin, embedded in paraffin, and sectioned. Thioflavin S staining was performed to visualize β‐sheet‐rich amyloid plaques, and immunohistochemistry was conducted using the 6E10 monoclonal antibody to detect Aβ plagues. Body weights were recorded daily throughout the study.

### 4.5 PK Studies

IV and PO PK in rodents were processed by Sai Life Sciences Pvt. Ltd using 8‐ to 10‐week‐old C57BL/6 male mice following a single intravenous administration at 1 mg/kg and oral administration at 10 mg/kg. A total of eighteen mice (n = 9/group) were divided into two groups as Group 1 and Group 2, with a sparse sampling design (3 mice/time points). Animals in Group 1 were administered intravenously as slow bolus injections through the tail vein at 1 mg/kg dose, and animals in Group 2 were administered through the oral route at 10 mg/kg. For 0043, the formulation vehicle used for IV group was 5% NMP, 10% PG, 5% Solutol HS‐15, and 80% Captisol (10% w/v in RO water), and the formulation used for the PO group was 10% NMP, 10% PG, 10% TPGS, and 70% Captisol (10% w/v in RO water). For 0152, the formulation vehicle used for IV group was 5% DMSO, 20% Solutol HS‐15, and 75% PBS, and the formulation used for PO group was 5% NMP, 10% PG, 5% Solutol HS‐15, and 80% Captisol (10% w/v in RO water). Blood samples (approximately 60 µL) were collected under light isoflurane anesthesia (Surgivet) from retro orbital plexus from a set of three mice at 0.08, 0.25, 0.5, 1, 2, 4, 6, 8 and 24 h for IV and PD, 0.5, 1, 2, 4, 6, 8 and 24 h for PO. Immediately after blood collection, plasma was harvested by centrifugation at 10 000 rpm, 10 min at 40°C, and samples were stored at ‐70±10°C until bioanalysis. All samples were processed for analysis by the protein precipitation method and analyzed with a fit‐for‐purpose LC‐MS/MS method (LLOQ = 1.01 ng/mL). The plasma pharmacokinetic parameters were estimated using the non‐compartmental analysis tool of Phoenix WinNonlin software (Ver 8.3).

### 4.6 Brain/Plasma(B/P) Ratio

Brain/Plasma Ratio test was processed by Sai Life Sciences Pvt. Ltd. Briefly, twelve male C57BL/6 mice were administered orally with solution formulation of 0152 at 10 mg/kg dose. Blood samples (approximately 60 µL) were collected under light isoflurane anesthesia from a set of three mice at 0.25, 0.5, 1, and 2 h. The blood samples were collected at each time point in labelled micro centrifuge tube containing K2EDTA as anticoagulant. Plasma was harvested by centrifugation of blood and stored at ‐70±10°C until analysis. Immediately after collecting blood, animals were anesthetized, perfused using 10 mL phosphate buffer saline, and brain samples were collected from each mouse at respective time points. Brain samples were homogenized using ice‐cold phosphate buffer saline (pH 7.4), and homogenates were stored below ‐70±10°C until analysis. Total homogenate volume was three times the brain weight. The plasma and brain concentration‐time data of 0152 were used for calculation. Plasma and brain samples were quantified by fit‐for‐purpose LC‐MS/MS method.

### 4.7 MDR1‐MDCKII Assay

MDR1‐MDCKII assay was processed by Sai Life Sciences Pvt. Ltd. Briefly, MDR1‐MDCKII cells were seeded onto the polycarbonate membranes in the 24‐well insert system at 0.12 × 106 cells/well and cultured for 8 days until confluence before being used for the transport studies. Test compounds were diluted with the transport buffer (HPSS with 10 mm HEPES, pH 7.4) from a DMSO stock solution to a concentration of 2 µm (DMSO < 1%) and applied to the apical or basolateral side of the cell monolayer. The plate was incubated for 2.0 h in a CO2 incubator at 37±1°C, with 5% CO2 at saturated humidity without shaking. Permeability was measured in duplicate (n = 2) in both A→B and B→A directions. Loperamide (P‐gp efflux substrate) was tested at 2 µm bidirectionally, Verapamil was used as P‐gp inhibitor. While Atenolol (low permeability marker) and Propranolol (high permeability marker) were tested at 2.00 µm in the A to B direction in duplicate. Test and reference compounds were quantified by LC‐MS/MS analysis based on the peak area ratio of analyte/internal standard (IS). The efflux ratio of each compound was calculated. Permeability measurements were performed in duplicate (n = 2) in both directions, and the summarized permeability values and efflux ratios are presented in Figure 3A. The corresponding experimental data, including bidirectional Papp values, recovery, and variability (%CV), are provided in Table S3, thereby supporting the results shown in Figure 3A. After the transport assay, Lucifer yellow fluorescence rejection assay was performed to confirm the integrity of the cell monolayer.

### 4.8 Kinetic Solubility Assay

10 µL of 10 mm DMSO stock solution of test and control compounds was added to 990 µL phosphate buffer with pH 7.4. The solubility samples were vortexed for at least 2 min, then shaken on a shaker at room temperature for 24 h. Samples were centrifuged, and the supernatant was transferred into a filter plate. Then, the filtrates were quantified by an LC‐MS system to get solubility results.

### 4.9 Cellular Thermal Shift Assay (CETSA)

For CETSA, as described previously [80], iPSC‐Ns or APP695WT‐HEK were treated in the presence or absence of 0043 at the saturating dose of 100 µm for 1 h to achieve detectable shifts in the assay, trypsinized, washed, and resuspended in 100 µL of PBS with protease inhibitors (600 000 cells/tube). Cells were incubated at their designated temperatures for 3 min, followed by 25°C for 3 min. Then, NP40 (Final 1%) was added to lyse the cells, followed by immediate snap freezing with liquid nitrogen and thawing using a thermal cycler at 25°C. This freezing and thawing cycle was repeated twice. The lysates were spun at 13 500 rpm for 20 min at 4°C, and the supernatant was harvested for western blotting analysis.

### 4.10 Thermofluor Assay

For the Thermofluor assay, Huynh's protocol [81] was used with minor modifications. In brief, all proteins were used at a final concentration of 5 µm for this assay, and GloMelt Thermal Shift Protein Stability Kit (Biotium) was used at a final concentration of 0.5X. All experiments were carried out with QuantStudio 6 Flex (Applied Biosystems). SYBR was used as a reporter, and ROX was used as a passive reference according to the manufacturer's directions. Melting curve data were exported, followed by analysis with Boltzmann's sigmoidal curve in GraphPad Prism.

### 4.11 Cytotoxicity and Cell Viability Assay

Cytotoxicity test was conducted using Neutral Red Uptake assay [82]. In brief, iPSC‐Ns seeded in 96 well plates (1 × 105 cells/well) were treated dose‐dependently for 5 days. Cells were washed with PBS containing CaCl2 and MgCl2 followed by the addition of Neutral red solution (40 µg/mL in PBS, Sigma–Aldrich) and incubation at 37°C for 3 h. Cells were then washed with PBS containing CaCl2 and MgCl2 two times and de‐stained using with Neutral red de‐staining solution on a plate shaker for 30 min, followed by reading at 540 nm with a Microplate reader. Cell viability was determined using the CellTiter‐Glo Luminescent Cell Viability Assay (Promega) following the manufacturer's instructions. In brief, HEK cells were plated at 5 × 104 cells/well, iPSC‐Ns at 1 × 105 cells/well in 96‐well plates and treated with drugs at 10 µm for 48 h.

### 4.12 Western Blotting and Immunoprecipitation

Cells were lysed in 1% Triton X‐100 buffer (50 mm Tris (pH 7.4), 150 mm NaCl, 10% glycerol, 1% Triton X‐100, 1 mm EDTA) or RIPA buffer (50 mm Tris (pH7.5), 150 mm NaCl, 0.5% NP40, 0.5% deoxycholate, 0.1% SDS) supplemented with 1 mm PMSF, protease and phosphatase inhibitors. For immunoprecipitation, cell lysates (0.5–1 mg) were incubated with the primary antibodies (1–2 µg) overnight at 4°C and mixed with protein G beads for 4 h. The immunoprecipitants were washed with 1% Triton X‐100 buffer 3 times and eluted with Laemmli buffer, followed by western blot. Immunoprecipitations were performed using the following approaches: (i) Tagged CAPRIN1 immunoprecipitation (HEK293 overexpression assays): HEK293 cells were co‐transfected with APP constructs (including APP695‐HiBiT) and Flag‐ or Myc‐tagged CAPRIN1. CAPRIN1 was immunoprecipitated using anti‐Flag or anti‐Myc antibodies, and co‐precipitated APP was detected by immunoblot; (ii) Endogenous APP immunoprecipitation: APP was immunoprecipitated using anti‐APP antibody (Sigma, A8717), followed by immunoblotting for CAPRIN1. Where indicated, reciprocal immunoprecipitation using anti‐CAPRIN1 antibody was performed, followed by APP detection; (iii) CAPRIN1 immunoprecipitation: In selected experiments, CAPRIN1 was immunoprecipitated using anti‐CAPRIN1 antibody (Proteintech, 15112‐1‐AP), and co‐precipitated proteins including APP and LAMP1 were analyzed by immunoblot; (iv) HiBiT‐based pull‐down assays: In HEK293 cells expressing APP695‐HiBiT, APP constructs were immunoprecipitated using anti‐HiBiT antibody (Promega, N7200), followed by detection of CAPRIN1. Western blot bands were quantified using Fiji (ImageJ) with background subtraction and normalized to loading controls. A complete list of antibodies used for western blotting, immunoprecipitation, and immunostaining is provided in Table S2.

### 4.13 Real‐Time PCR

Total RNA was isolated using the RNeasy kit (Qiagen), followed by synthesis for cDNA with a reverse transcription reaction (Quantitect Reverse Transcription kit). The sequences for APP and GAPDH are in Table S1. Quantitative PCR was performed on QuantStudio 6 Flex (Applied Biosystems). The expression levels of APP were normalized to the GAPDH mRNA levels and represented relative to the expression in control cells. The results were analyzed with the delta‐delta‐Ct methods. Ct values were calculated using QuantStudio Design & Analysis Software, and relative expression levels were analyzed with GraphPad Prism.

### 4.14 Enzyme‐Linked Immunosorbent Assay (ELISA)

iPSC‐Ns or HEK‐APP695WT were treated with drugs, and secreted Aβ42 peptides inculture soup were quantified using the Human Amyloid β1‐42 human ELISA kit (Thermo Fisher) according to the manufacturer's protocol. Mouse brain extraction was performed as previously described [83], with minor modifications. Mouse brain tissues were homogenized at a 1:10 weight‐to‐volume ratio in Tris‐buffered saline (TBS; 50 mm Tris, pH 7.4, 150 mm NaCl) supplemented with 2 mm EDTA and protease inhibitors. TBS‐homogenized brains were centrifuged for 1 h at 16 000 g. To extract total Aβ, including insoluble fractions, formic acid was added to a final concentration of 70%, and the samples were thoroughly mixed by pipetting. Homogenates were centrifuged at 100 000 g for 1 h at 4°C, and the resulting supernatants were collected. Prior to quantification, formic acid‐containing samples were neutralized with 1 m Tris base (pH 11). Aβ40 and Aβ42 levels were measured using human‐specific ELISA kits (Invitrogen) and normalized to total protein concentrations determined by the BCA assay (Thermo Scientific). For Aβ quantification in 3D organoids, 3D organoids’ processing and ELISA analysis were performed with minor modifications from previously described protocols [84]. Briefly, cerebral organoids were lysed in RIPA buffer supplemented with protease and phosphatase inhibitor cocktails (Sigma), followed by sonication and incubation on ice for 60 min. Lysates were centrifuged at 13 000 rpm for 1 h at 4°C, and the supernatants were collected as the RIPA‐soluble fraction. The remaining pellets were resuspended in 70% formic acid, sonicated, and subsequently neutralized with Tris buffer to obtain the formic acid‐solubilized fraction. Aβ42 levels in both fractions were measured using human‐specific ELISA kits (Thermo Fisher) according to the manufacturer's instructions. Formic acid extracts were appropriately neutralized prior to ELISA. Aβ levels were normalized to total protein concentration determined by BCA assay.

### 4.15 Immunofluorescence Staining

Cells were grown on glass cover slips coated with 1% Matrigel and treated with drugs. Cells were washed, fixed, permeabilized, blocked, and incubated in primary antibodies diluted in blocking solution (1% v/v BSA solution/5% Goat serum in PBS containing 0.1% Triton X‐100) overnight at 4°C. The cells were washed three times with PBS and incubated in secondary antibodies in a blocking solution for 1 h at room temperature, then washed with PBS, followed by HOECHST staining (Invitrogen) for 5 min, and washed with PBS. The glass coverslips were put on a slide‐in mounting medium (VECTASHIELD), and the edges were sealed with coverslip sealant (BIOTIUM). Image data were captured and processed with a confocal microscope system (Olympus Fluoview FV1000 or Leica DIVE) and the ImageJ program. For 3D organoids staining, organoids were fixed in 10% formalin overnight at room temperature, followed by incubation in 30% sucrose solution overnight at room temperature, embedded in optimal cutting temperature compound (OCT, FisherScientific), and kept at ‐80°C overnight. Frozen samples were sectioned at 10 µm using a cryostat (Leica) and mounted on microscope slides (Fisherbrand superfrost plus, FisherScientific). Before immunolabeling, sections were rinsed in PBS for 5 min, permeabilized for 30 min in PBS containing 0.3% Triton‐X 100, blocked for 1 h in PBS containing 5% BSA and 0.1% Triton‐X 100, and incubated with primary antibodies diluted in the same blocking solution. Then, secondary staining, mounting, and imaging were followed.

### 4.16 Immunohistochemistry of Organoids, AD Patient Brains, and Mouse Brains

Autopsy samples of brain tissues were provided, and immunohistochemistry was performed in the Department of Pathology and Laboratory Medicine, Indiana University School of Medicine, following the institutional protocols. Paraffin‐embedded 3D organoids and human AD patient brain samples were subjected to Hematoxylin and Eosin (H&E), synapses, GFAP, CAPRIN1 (polyclonal Rabbit anti‐CAPRIN1, 15112‐1‐AP, Proteintech), APP (monoclonal mouse anti‐APP/Aβ (6E10), 803001, Biolegend; MAB348, Millipore Sigma) in a Dako automated instrument using the Dako Flex detection system. The primary antibodies and secondary antibodies were used in colocalization experiments with human AD brain biopsy samples, then imaged using a confocal microscope system. Thioflavin‐S was stained with deparaffinization and rehydration of mouse brain sections using a series of xylene and ethanol washes. The sections were then incubated in filtered 1% aqueous Thioflavin‐S for 8 min at room temperature, protected from light, followed by sequential washes in ethanol and distilled water. Finally, slides were covered with aqueous mounting solution, dried overnight in the dark, sealed with nail polish, and stored at 4°C to prevent fading before analysis.

### 4.17 Imaging Analysis

ImageJ (Fiji) program (NIH, Bethesda, MD, USA) was used o analyze images [85]. Pearson's correlation coefficients were calculated with the JACoP plugin from two channels (BIOP). For colocalization/trafficking analysis with organelles in iPSC‐Ns, HEK293 cells, and 3D organoids, APP or CAPRIN1 with organelles signals were segmented using a trainable weka segmentation plugin in Fiji as previously described [86]. Colocalized areas were calculated in the image calculator tool (‘Or’ function) to get an overlayed area for the segmented signals, followed by using the analyze particle tool in Fiji to determine by > 1‐pixel overlapping of the segmented signal. To determine the fluorescence intensity, the corrected total cell fluorescence (CTCF) was used using the formula: CTCF = Integrated Density—(Area of selected cell x Mean fluorescence of background readings), as described previously [87]. Bright‐field images of immunoperoxidase‐stained and Thioflavin S‐stained brain sections were exported as 16‐bit RGB TIFF files and analyzed using ImageJ (Fiji), following previously established protocols [88, 89]. To isolate the DAB signal for quantification, color deconvolution was performed using the “H DAB” setting in the Color Deconvolution plugin in ImageJ. The resulting “Colour_2” image, corresponding to the DAB chromogen, was converted to 8‐bit grayscale for further analysis. Manual thresholding was applied to the DAB channel using the threshold adjustment tool in ImageJ. Threshold levels were individually adjusted for each image to match the visual intensity of the stained signal, and the threshold image was displayed in black and white for clarity. Regions of interest (ROIs), specifically the subiculum, hippocampus, and cortex, were manually delineated based on anatomical landmarks and saved using the ROI Manager. For each ROI, the DAB‐positive area was quantified using the Analyze Particles plugin with default particle size settings. The percentage of DAB‐positive area was calculated as the ratio of the DAB‐positive area to the total area of each ROI.

### 4.18 CAPRIN1‐APP Interaction by Glycerol Gradient

About 1 mg of 10xHis‐MBP‐APP695 was mixed with ∼1 mg of 10xHis‐CAPRIN1 and loaded onto 10%–30% (v/v) glycerol gradient containing the buffer (50 mm Hepes‐KOH pH7.6, 200 mm KCl, 5 mm β‐mercaptoethanol, and 0.1% DDM). For controls, ∼1 mg of 10xHis‐MBP‐APP695 (full‐length) alone was loaded onto 10%–30% (v/v) glycerol gradient as well. After centrifugation at 35 000 rpm in a Beckman SW Ti‐40 rotor for 19 h at 4°C, the gradient was fractionated as described above. For fractions containing 10xHis‐CAPRIN1, a PGF Piston Gradient Fractionator was used. Fractions containing 10xHis‐CAPRIN1, 10xHis‐MBP‐APP695 alone or a mixture were subjected to 4%–12% NuPAGE (Invitrogen) and stained with Coomassie blue.

### 4.19 In Vitro Binding Assay

When CAPRIN1 was used as a bait, recombinant CAPRIN1 (200 ng) was mixed with anti‐CAPRIN1 antibodies (1 µg) in IP buffer (25 mm Tris, 150 mm NaCl, 5% Glycerol, and 1% Triton X‐100 at pH 7.4) overnight at 4°C, followed by the addition of Protein‐G agarose beads for 4 h at 4°C. CAPRIN1 conjugated beads were then washed with binding buffer (20 mm HEPES, 100 mm NaCl, 0.01% Triron X‐100, and 5% Glycerol at pH 7.25) before the addition of recombinant human APP (prey) for 1 h at 4°C. Beads were washed and eluted with Laemmli buffer with boiling, followed by Western blotting. When APP was used as a bait, biotin‐conjugated APP antibody (1 µg; 4G8, Biolegend) was pre‐mixed with recombinant APP (100 ng, Biolegend) in pulldown buffer (0.1% NP‐40, 50 mm Tris, 150 mm NaCl, 0.2 mm EDTA, 100 mm KCl, 1 mm MgCl2, 0.2 mm CaCl2, and 10% glycerol) overnight at 4°C, followed by addition of 0.5 mg of Streptavidin magnetic beads (Pierce) for 1 h at 4°C. After washing the beads, recombinant human CAPRIN1 (full length, 100 ng) was added together with dose‐dependent 0043 in the binding buffer for 1 h at 4°C. Beads were washed and eluted with Laemmli buffer with boiling, followed by Western blotting.

### 4.20 Surface Plasmon Resonance (SPR)

SPR experiments were conducted to investigate the interaction between APP695 and CAPRIN1 proteins using a Biacore T200 instrument (Cytiva). Both proteins were expressed in Tni (Hi5) insect cells and purified to homogeneity via tandem affinity and ion‐exchange. Protein purity and integrity were confirmed by SDS‐PAGE and size‐exclusion chromatography prior to analysis. All experiments were performed in a running buffer consisting of 50 mm HEPES at pH7.0, 100 mm KCl, 10% glycerol, and 0.01% (v/v) Tween‐20 supplemented with 0.1% (w/v) Sarkosyl (pH 7.4). APP695 was serially diluted in running buffer to final concentrations of 15, 5, 1, and 0.3 µm. Chip Preparation: A CM5 sensor chip (Cytiva) was activated using a 1:1 (v/v) mixture of 0.1 m N‐hydroxysuccinimide (NHS) and 0.1 m N‐ethyl‐N′‐(3‐dimethylaminopropyl) carbodiimide (EDC) to generate reactive ester groups. CAPRIN1 was diluted to 50 µg/mL in 10 mm Hepes‐KOH buffer (pH 6.0) and immobilized on the chip surface via standard amine coupling to achieve immobilization. Unreacted sites were blocked with 1 m ethanolamine‐HCl (pH 8.5). A reference flow cell was prepared similarly but without protein immobilization to control for non‐specific binding and bulk refractive index changes. Binding assay: Binding interactions were measured at 25°C with a flow rate of 30 µL/min. APP695 solutions were injected over the CAPRIN1‐immobilized and reference flow cells for 45 s (association phase), followed by a 240 s dissociation phase with running buffer alone. Internal control injections for one concentration of APP695 were performed to ensure reproducibility for every round of the experiment. 0043 and 0152 produced no noticeable interaction with CAPRIN1 alone. APP (1–300) was also tested for binding to CAPRIN1 under identical conditions. To assess potential non‐specific interactions, 0043 and 0152 were tested for binding to APP‐695 captured on a CM5 chip using a steady‐state model. Data analysis: Sensograms were processed using Biacore T200 Evaluation Software. Signals from the reference flow cell were subtracted from those of the CAPRIN1 flow cell to account for non‐specific binding and bulk refractive index effects. Baseline drift was corrected by subtracting responses from blank buffer injections. Binding data were globally fitted to a heterogeneous ligand model to calculate kinetic parameters, including the association rate constant (kon) and dissociation rate constant (koff). Equilibrium dissociation constants (KD) were derived from the ratio koff/kon.

### 4.21 Human Brain Proteomic Analysis

Human brain proteomic data were accessed through AGORA (Alzheimer's Disease Target Discovery Platform), which provides access to multi‐omic datasets generated by the Accelerating Medicines Partnership‐Alzheimer's Disease (AMP‐AD) consortium. Differential protein expression of APP and CAPRIN1 was analyzed using the dorsolateral prefrontal cortex (DLPFC) tandem mass tag (TMT) proteomic dataset available through AGORA. Log2 fold change (log2FC) and adjusted *p* values were obtained directly from the platform. AGORA is available at doi:10.57718/agora‐adknowledgeportal.

### 4.22 Statistical Analysis

Statistical analyses were performed using GraphPad Prism 9.0 software. Data are presented as mean ± standard deviation (SD), and the number of biological replicates (n) is indicated in each figure legend. Comparisons between two groups were analyzed using unpaired two‐tailed Student's *t*‐tests. For comparisons among three or more groups, one‐way analysis of variance (ANOVA) followed by Tukey's multiple comparisons test was used. For experiments involving two independent variables, two‐way ANOVA with appropriate post hoc tests (Tukey's or Bonferroni's) was applied. Dose–response curves and IC_50_ values were generated using nonlinear regression with a three‐parameter variable slope model. For thermal shift assays (CETSA and ThermoFluor), melting curves were fitted using Boltzmann's sigmoidal function. Pearson's correlation coefficients were calculated for confocal image colocalization analysis using the JACoP plugin in ImageJ (Fiji). Pharmacokinetic parameters were estimated using non‐compartmental analysis in Phoenix WinNonlin v8.3. Statistical significance was defined as *p* < 0.05 and denoted as follows: *p* < 0.05 (^*^), *p* < 0.01 (^**^), *p* < 0.001 (^***^), and *p* < 0.0001 (^****^); “ns” indicates not significant.

## Author Contributions

C.H., A.C.B., and A.S. initiated this APP drug discovery project. S.H.J., A.C.B., and C.H. designed the experiments and provided supervision. S.H.J. carried out all the drug screenings, molecular, biochemical, iPSC experiments, and animal studies. X.W., B.L., C.G., L.Z., and H.Y.L. designed, synthesized, and analyzed compounds. R.R., N.J., R.K.R., Y.T. R.K.H. expressed and purified recombinant proteins. R.R. designed and performed structural studies. N.J. designed and carried out recombinant protein interaction. R.K.W. assisted with biological work. B.T.L. supervised AD mouse model studies. S.H.J., A.C.B. and C.H. analyzed and organized all the data and co‐wrote the manuscript. All authors reviewed and contributed to editing the manuscript.

## Funding

This work was supported in part by the National Institute on Aging (R41 AG090241, C.H.) and the National Institute of Neurological Disorders and Stroke (R01 NS126358, C.H.). C.H. was supported in part by the endowment fund of the Bicentennial Chair at the Indiana University School of Medicine.

## Ethics Statement

All mouse studies were conducted in accordance with protocols approved by the Institutional Animal Care and Use Committee.

## Conflicts of Interest

A.C.B. and C.H. are co‐founders of Degrome Therapeutics, Inc. and HB Therapeutics, Inc. H.Y.L. is an employee of Synovel Laboratory. The other authors declare no competing interest. C.H., A.C.B., S.H.J., C.G., L.Z., and X.W. are co‐inventors on a patent application (PCT/US24/60393), titled “Compounds and methods to treat Alzheimer's disease,” filed by the Indiana University Innovation & Commercialization Office.

## Supporting information




**Supporting File**: advs77575‐sup‐0001‐SuppMat.pdf.

## Data Availability

All data supporting the finding of this study are included in the main test and supplementary materials. The compounds will be made available to academic researchers through a material transfer agreement upon request to the correspondent authors.
