# Supplementary material for: Molecular Glue Degraders Enhance CAPRIN1‐Dependent Lysosomal Degradation of APP and Reduce Amyloid β in Alzheimer's Disease
Source: Adv Sci (Weinh). 2026 Sep 1:e77575. Online ahead of print. doi: 10.1002/advs.77575 (PMC13534789; doi:10.1002/advs.77575)
Supplement: Supplementary file 1 [file ADVS-9999-e77575-s001.pdf]

Supplementary Materials for

Molecular glue degraders enhance CAPRIN1-dependent lysosomal degradation of APP and reduce amyloid β in Alzheimer's disease

Sunghan Jung, Xu Wang, Bin Liu, Raktim Roy, Nancy Jaiswal, Ratan K. Rai, Yuichiro Takagi, Cen Gao, Lifan Zeng, Ho-Yin Lo, Reagan K. Wohlford, Ryan K. Higgins, Bruce T. Lamb, Anantha Shekhar, Anita C. Bellail, Chunhai Hao.

*Corresponding authors:
Sunghan Jung, jung14@iu.edu
Anita C. Bellail, abellail@iu.edu
Chunhai Hao, chunhao@iu.edu

This PDF file includes:

Figure. S1 to S7
Tables S1 to S3
Supplemental Materials and Methods

Figure S1

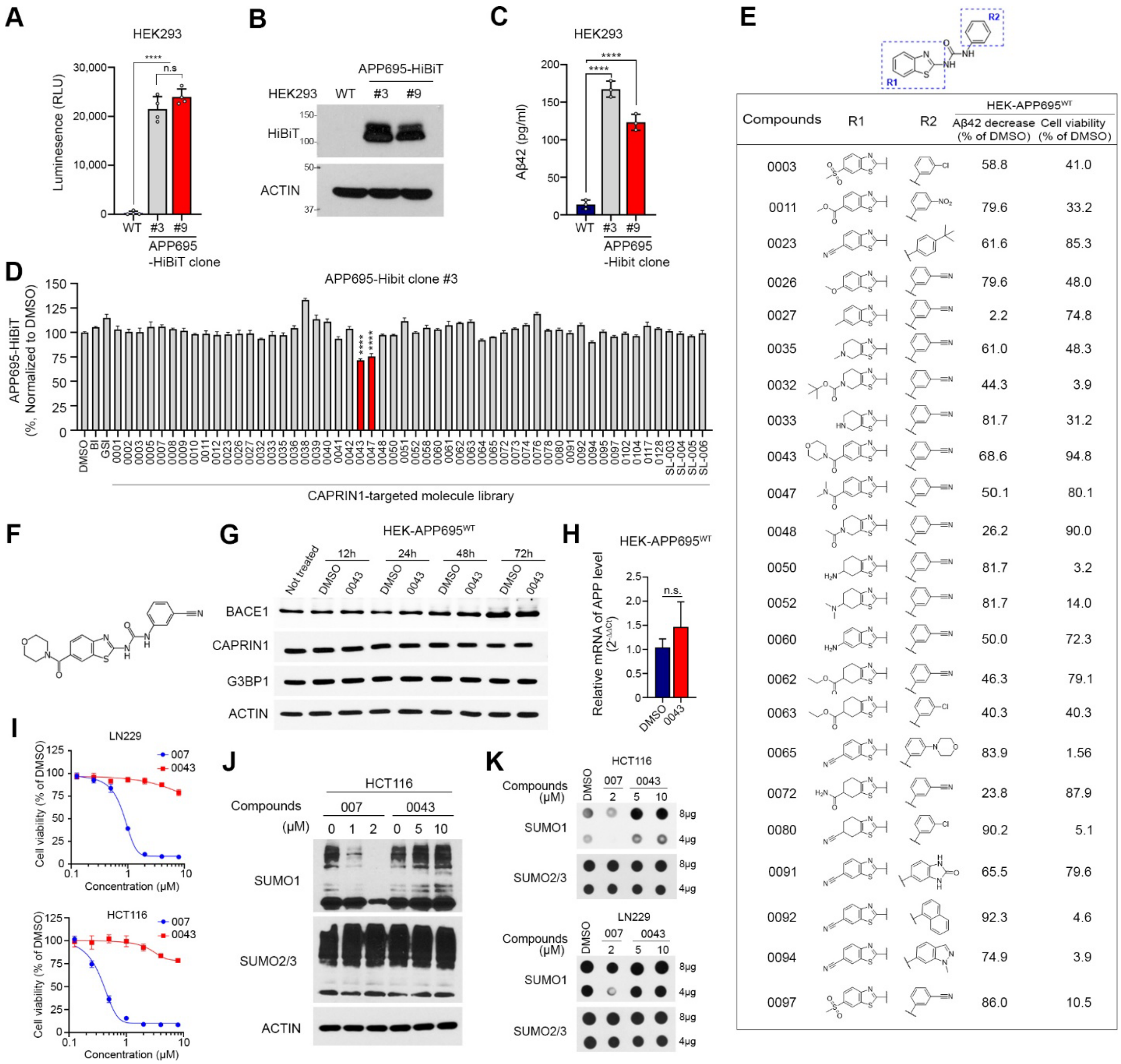
A
HEK293
Luminesence (RLU)
WT #3 #9
APP695 -HiBiT clone
B
APP695-HiBiT
HEK293 WT #3 #9
HiBiT
ACTIN
C
HEK293
Aβ42 (pg/ml)
WT #3 #9
APP695 -Hibit clone
D
APP695-Hibit clone #3
APP695-HiBiT (%, Normalized to DMSO)
CAPRIN1-targeted molecule library
E
Compounds R1 R2 HEK-APP695WT Aβ42 decrease (% of DMSO) Cell viability (% of DMSO)
0003 58.8 41.0
0011 79.6 33.2
0023 61.6 85.3
0026 79.6 48.0
0027 2.2 74.8
0035 61.0 48.3
0032 44.3 3.9
0033 81.7 31.2
0043 68.6 94.8
0047 50.1 80.1
0048 26.2 90.0
0050 81.7 3.2
0052 81.7 14.0
0060 50.0 72.3
0062 46.3 79.1
0063 40.3 40.3
0065 83.9 1.56
0072 23.8 87.9
0080 90.2 5.1
0091 65.5 79.6
0092 92.3 4.6
0094 74.9 3.9
0097 86.0 10.5
F
G
HEK-APP695WT
Not treated 12h 24h 48h 72h DMSO 0043
BACE1
CAPRIN1
G3BP1
ACTIN
H
HEK-APP695WT
Relative mRNA of APP level
n.s.
DMSO 0043
I
LN229
007
0043
Cell viability (% of DMSO)
Concentration (μM)
HCT116
J
HCT116
Compounds 007 0043
(μM) 0 1 2 0 5 10
SUMO1
SUMO2/3
ACTIN
K
HCT116
Compounds (μM) DMSO 007 2 0043 5 10
SUMO1
SUMO2/3
8μg
4μg
LN229

Figure. S1. Cell-based screening identifies CAPRIN1-targeted APP degraders.
(**A**) HiBiT luminescence signal in HEK293 cells stably expressing APP695-HiBiT clones (#3, #9) via lentiviral transduction (mean ± s.d., n = 4; ****p < 0.0001). (**B**) Western blot showing APP695-HiBiT expression in selected HEK clones (#3, #9). (**C**) Aβ42 levels in culture media of HEK cells expressing WT, #3, or #9 APP695–HiBiT measured by ELISA (mean ± s.d., n = 3; ****p < 0.0001). (**D**) Compound screening in HEK–APP695^{WT} cells expressing APP695-HiBiT clone #3 from 10 μM CAPRIN1-targeted small molecules for 24 h; changes shown as % of DMSO control, highlighting compounds 0043 and 0047 in red (–****p < 0.0001). (**E**) Chemical structures and summary table of selected compounds from the library screen, showing Aβ42 reduction and cell viability relative to DMSO. (**F**) Chemical structure of compound 0043. (**G**) Time-course western blot of BACE1, CAPRIN1, and G3BP1 in HEK-APP695WT cells treated with 0043 (10 μM) for 12, 24, 48, or 72 h. (**H**) qRT-PCR quantification of APP mRNA in HEK-APP695^{WT} cells treated with DMSO or 0043 for 72 h (mean ± s.d., n = 3; n.s., not significant). (**I**) Cell viability of LN229 and HC116 treated with a series of dilutions of HB007 and 0043 (means ± SD, N=3). (**J**) Western blots of SUMO1 levels in HCT116 cells treated with indicated doses of HB007 and 0043. SUMO2/3 and actin were used as the loading controls. (**K**) Dot blots of SUMO1 levels in HCT116 and LN229 cells treated with indicated doses of HB007 and 0043 for 3 days.

Figure S2

A

Cell	Diagnosis	Gene	Mutation	APOE Genotype	Sex
sAD2.3	sAD	-	None reported	Unknown	Male
AG27606	AD	-	None reported	Unknown	Male
AG27608	AD	-	None reported	Unknown	Male
UKBi011A	AD	APOE	None reported	E4/E4	Male
AG27609	AD	APOE	None reported	E4/E4	Anonymized
AG25367	fAD	PSEN1	p.Ala246Glu	Unknown	Female
AG25370	fAD	PSEN2	p.Asn141Ile	Unknown	Female
HVRDi002A	fAD	APP	V717I	Unknown	Female
AG28262	Non-AD	-	None reported	Unknown	Female
AG28049	Non-AD	-	None reported	Unknown	Male
AG27602	Non-AD	-	None reported	Unknown	Male

B

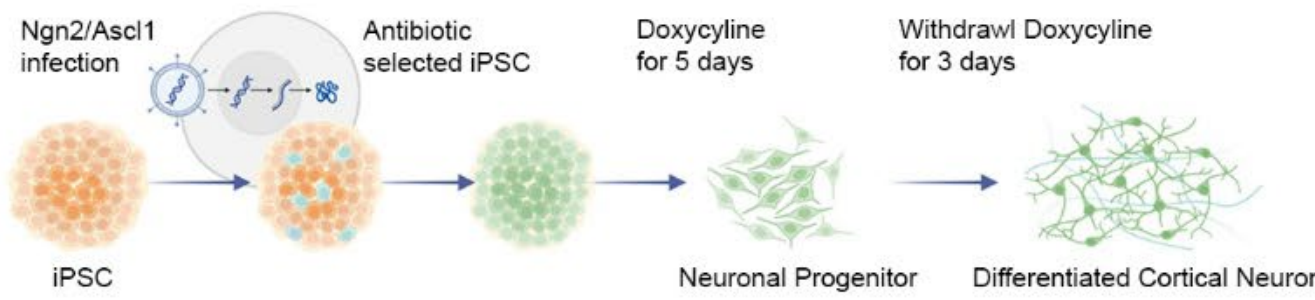

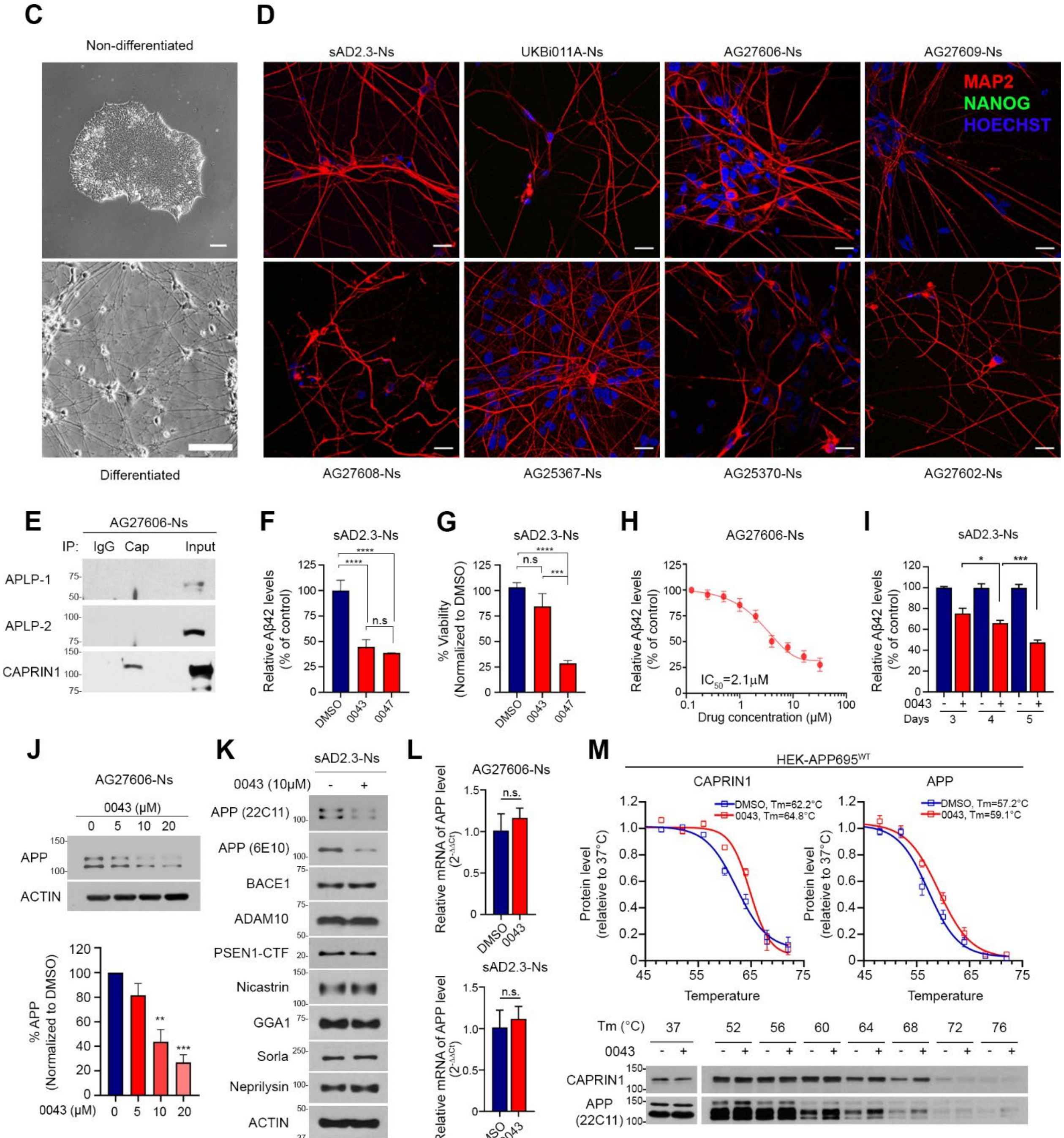

Figure. S2. The hit compound 0043 reduces APP and Aβ42 in AD iPSC-derived neurons. (**A**) Table summarizing diagnosis, genotype, and sex of iPSC lines used. (**B**) Schematic of Ngn2/Ascl1-based cortical neuron differentiation from iPSCs using doxycycline-inducible system. (**C**) Representative phase-contrast images before and after differentiation (scale bars, 100 µm). (**D**) Confocal images of differentiated neurons stained for MAP2 (red), NANOG (green), and Hoechst (blue) (scale bars, 20 µm). (**E**) CAPRIN1 IP in AG27606-Ns, followed by western blot of APLP1/2. (**F**) Aβ42 levels in culture media of sAD2.3 neurons treated with DMSO, 0043, or 0047 (10 µM, 72 h) by ELISA (mean ± s.d., n = 3; ****$P < 0.0001$; n.s., not significant). (**G**) Cell viability of sAD2.3 neurons after 72 h treatment with compounds measured by CellTiter-Glo (mean ± s.d., n = 3). (**H**) Dose-response curve of Aβ42 reduction in AG27606-Ns treated with 0043 for 5 days (IC_{50} = 2.1 µM; mean ± s.d.). (**I**) Time-course analysis of Aβ42 in sAD2.3 neurons treated with 0043 (10 µM) for 3–5 days (mean ± s.d.; *$p < 0.05$, **$p < 0.01$). (**J**-**K**) Western blots of APP and related proteins in AG27606-Ns (J) and sAD2.3-Ns (K) treated with 0043 (10 µM, 72 h, n = 3). (**L**) qRT-PCR quantification of APP mRNA in AG27606-Ns and sAD2.3 neurons treated with DMSO or 0043 for 72 h (mean ± s.d., n = 3; n.s., not significant). (**M**) Cellular thermal shift assay (CETSA) in HEK-APP695^{WT} cells treated with DMSO or 0043 (10 µM, 1 h), showing thermal stability curves and representative western blots; apparent melting temperatures (Tm) indicated.

Figure S3

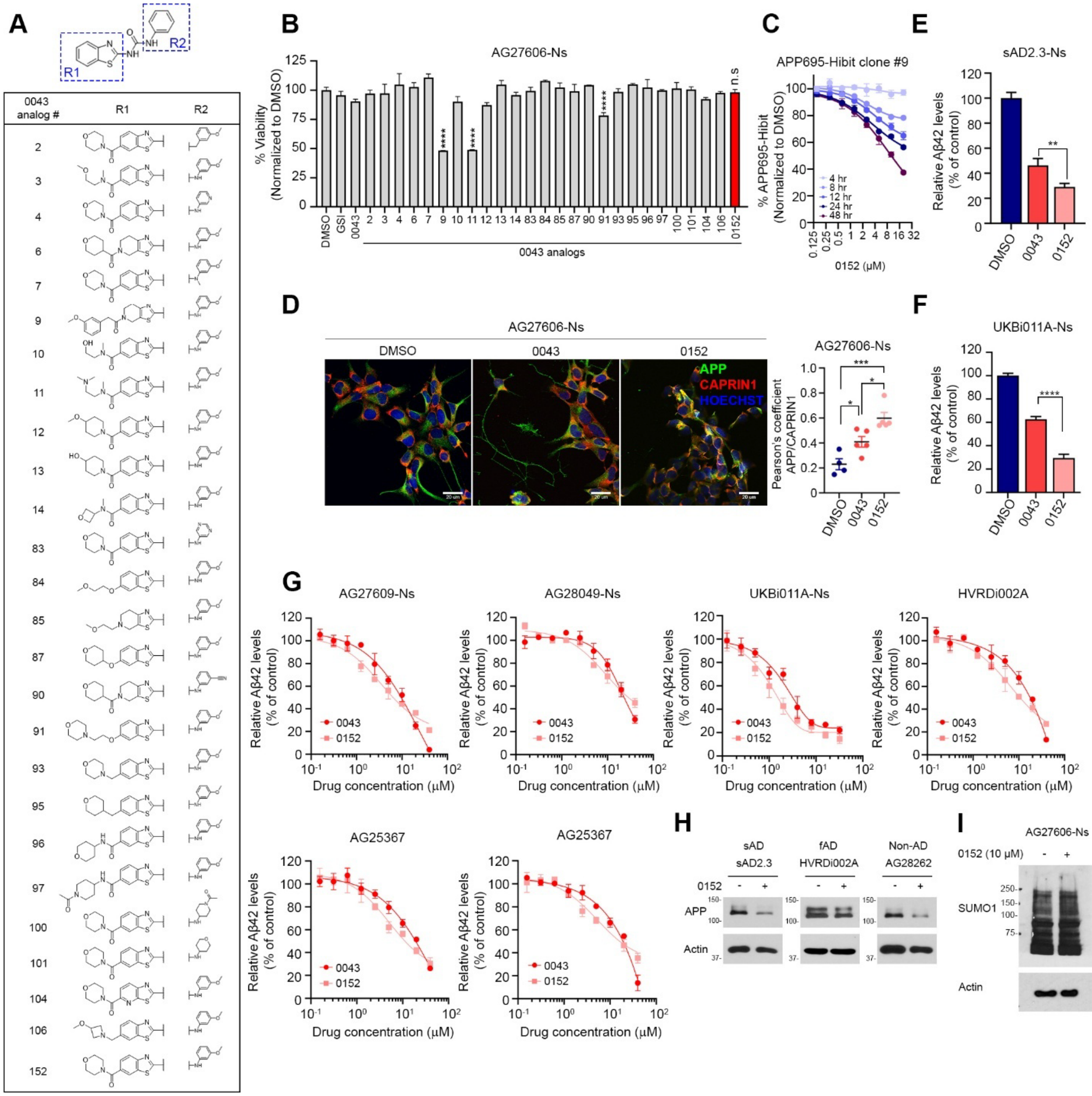
A
R1
R2
0043 analog #
B
AG27606-Ns
% Viability (Normalized to DMSO)
0043 analogs
C
APP695-Hibit clone #9
% APP695-Hibit (Normalized to DMSO)
0152 (μM)
D
AG27606-Ns
DMSO
0043
0152
APP
CAPRIN1
HOECHST
Pearson's coefficient APP/CAPRIN1
E
sAD2.3-Ns
Relative Aβ42 levels (% of control)
F
UKBi011A-Ns
G
AG27609-Ns
AG28049-Ns
UKBi011A-Ns
HVRDi002A
AG25367
Drug concentration (μM)
H
sAD sAD2.3
fAD HVRDi002A
Non-AD AG28262
APP
Actin
I
AG27606-Ns
0152 (10 μM)
SUMO1
Actin

Figure. S3. Optimization of 0043 derivatives identifies the lead compound 0152.

(**A**) Chemical structures of 0043 analogs; table summarizes structural diversity at R1 and R2 positions. (**B**) Cell viability of AG27606-Ns treated with 0043 and 20 analogs (10 μM, 72 h) measured by CellTiter-Glo (mean ± s.d., n = 3). (**C**) Dose- and time-dependent reduction of APP695-HiBiT levels by compound 0152. (**D**) Confocal images of AG27606-Ns treated with DMSO, 0043, or 0152 (10 μM, 24 h), stained for APP (green), CAPRIN1 (red), and Hoechst (blue); scale bars, 20 μm. APP-LAMP1 colocalization quantified (mean ± s.d., n ≥ 4; one-way ANOVA with Tukey's). (**E,F**) Aβ42 levels in culture media of sAD2.3-Ns (E) and UKBi011A-Ns (F) treated with DMSO, 0043, or 0152 (10 μM, 72 h) by ELISA (mean ± s.d., n = 3; **p < 0.01, ****p < 0.0001). (**G**) Dose-response curves of Aβ42 reduction with 0043 or 0152 treatment for 5 days. All values are normalized to the DMSO-only control (0.1% DMSO), corresponding to the 0 μM condition. (**H**) Western blot of APP in iPSC-derived neurons from sAD, fAD, and non-AD donors treated with 0152 (10 μM, 72 h). **(I)** SUMO1 levels in AG27606-derived neurons treated with 0152 (10 μM, 3 days).

Figure S4

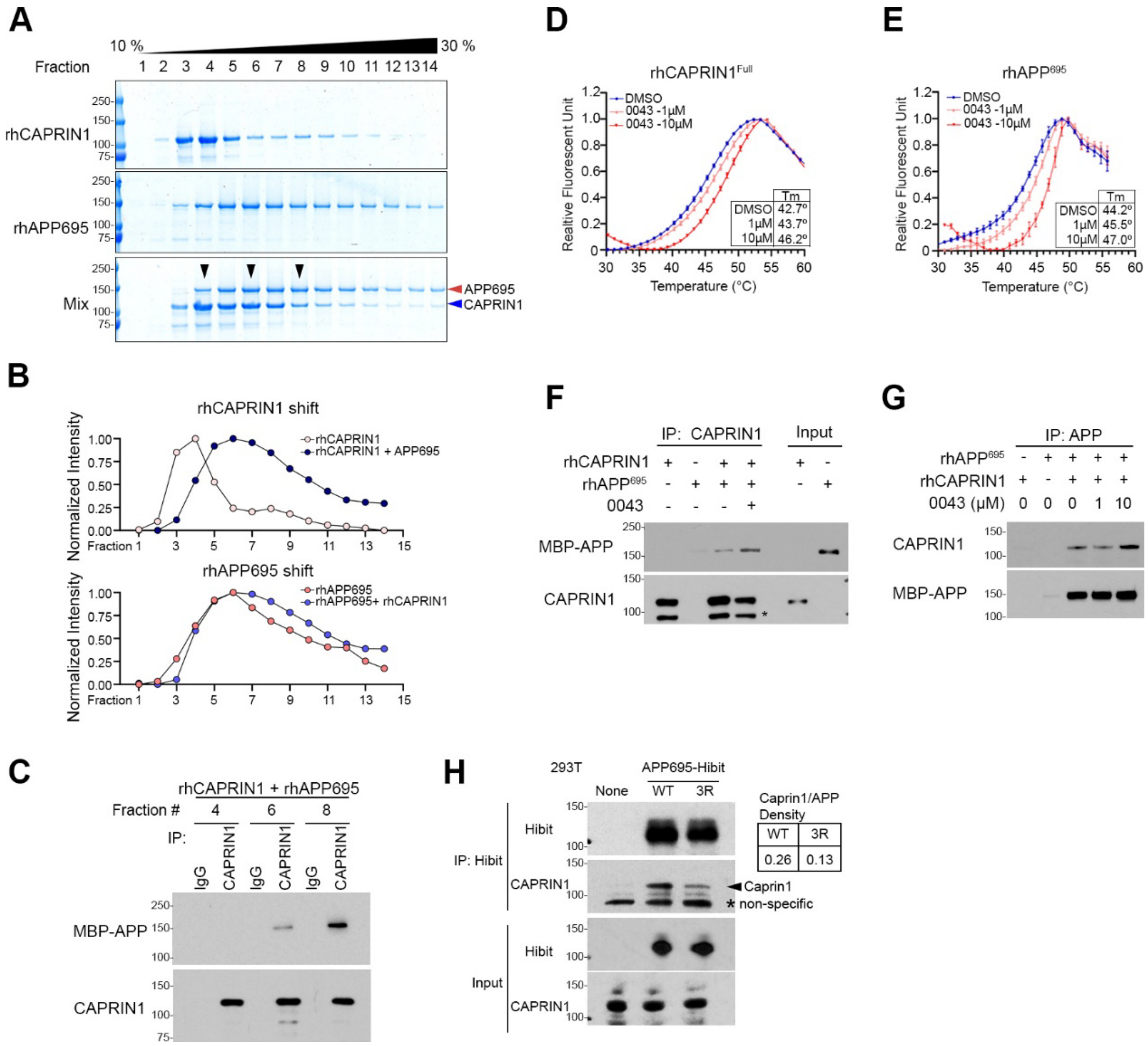

Figure. S4. Glue compounds enhance in CAPRIN1-APP interaction.
(**A**) SDS-PAGE analysis of fractions from glycerol gradient centrifugation of recombinant human CAPRIN1 (rhCAPRIN1), APP695 (rhAPP695), and their mixture; Arrowheads indicate fractions showing mobility shifts in the mixture. (**B**) Quantification of protein distribution across gradient fractions showing elution shifts of rhCAPRIN1 (top) and rhAPP695 (bottom). (**C**) Recombinant CAPRIN1 was immunoprecipitated from SEC fractions, and co-precipitated APP was detected by immunoblot. (**D,E**) Thermofluor assay melt curves of recombinant full-length CAPRIN1 (D) and APP (E) pre-incubated with 0043 or DMSO; protein stability quantified by luminescence across temperature gradients (mean ± s.d., n = 3). (**F,G**) Recombinant CAPRIN1 (F) or APP (G) was immunoprecipitated, and co-precipitated APP or CAPRIN1, respectively, was detected by immunoblot (0043 = 10 μM). **(H)** HiBiT-tagged APP (WT or 3R) was immunoprecipitated using an anti-HiBiT antibody, and co-precipitated CAPRIN1 was detected by immunoblot.

Figure S5

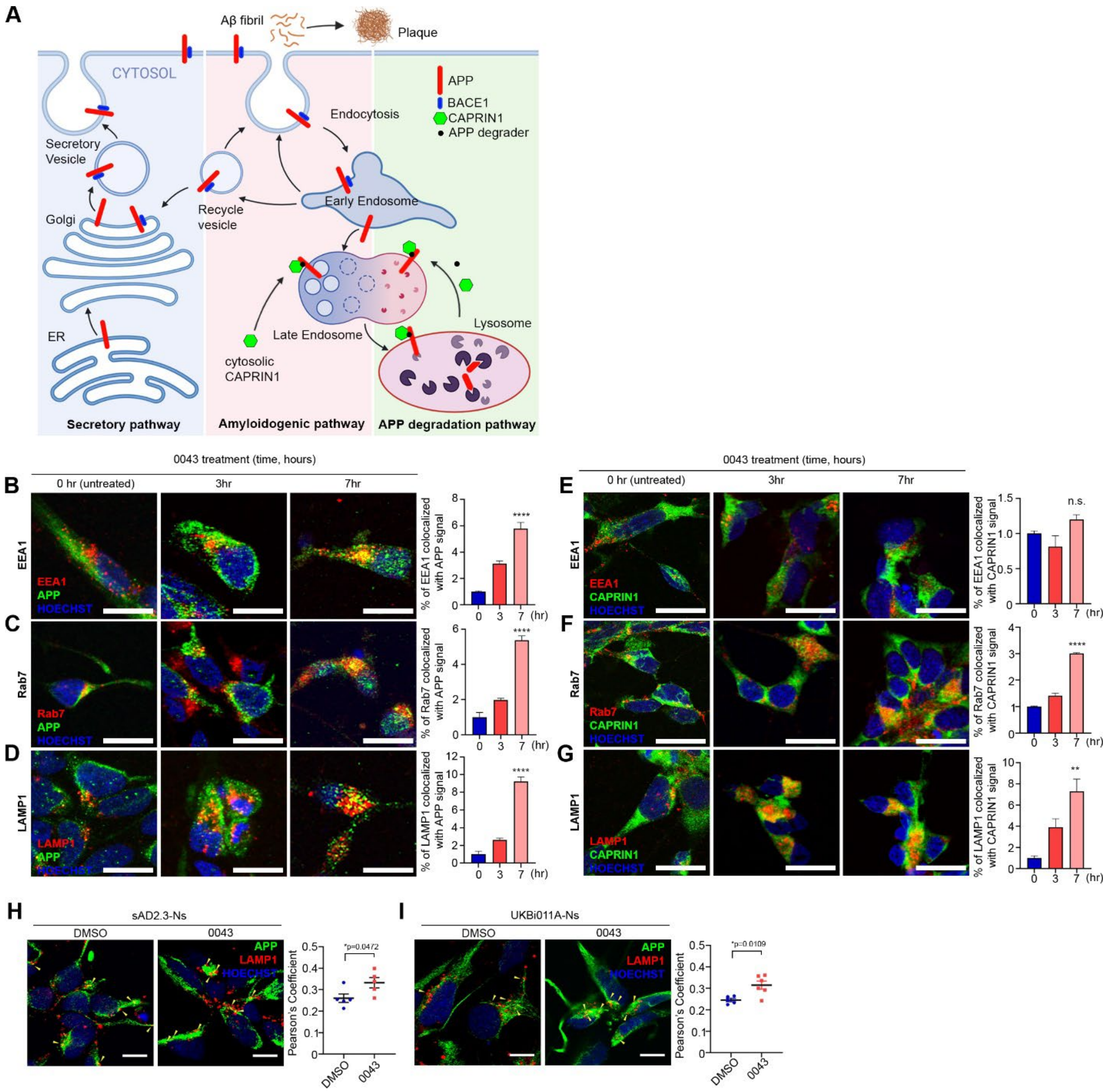

A
Aβ fibril
Plaque
CYTOSOL
APP
BACE1
CAPRIN1
APP degrader
Endocytosis
Secretory Vesicle
Golgi
Recycle vesicle
Early Endosome
Late Endosome
Lysosome
cytosolic CAPRIN1
ER
Secretory pathway
Amyloidogenic pathway
APP degradation pathway
0043 treatment (time, hours)
0 hr (untreated)
3hr
7hr
B
C
D
E
F
G
EEA1
Rab7
LAMP1
APP
CAPRIN1
HOECHST
% of EEA1 colocalized with APP signal
% of Rab7 colocalized with APP signal
% of LAMP1 colocalized with APP signal
% of EEA1 colocalized with CAPRIN1 signal
% of Rab7 colocalized with CAPRIN1 signal
% of LAMP1 colocalized with CAPRIN1 signal
****
n.s.
**
(hr)
H
sAD2.3-Ns
I
UKBi011A-Ns
DMSO
0043
Pearson's Coefficient
*p=0.0472
*p=0.0109

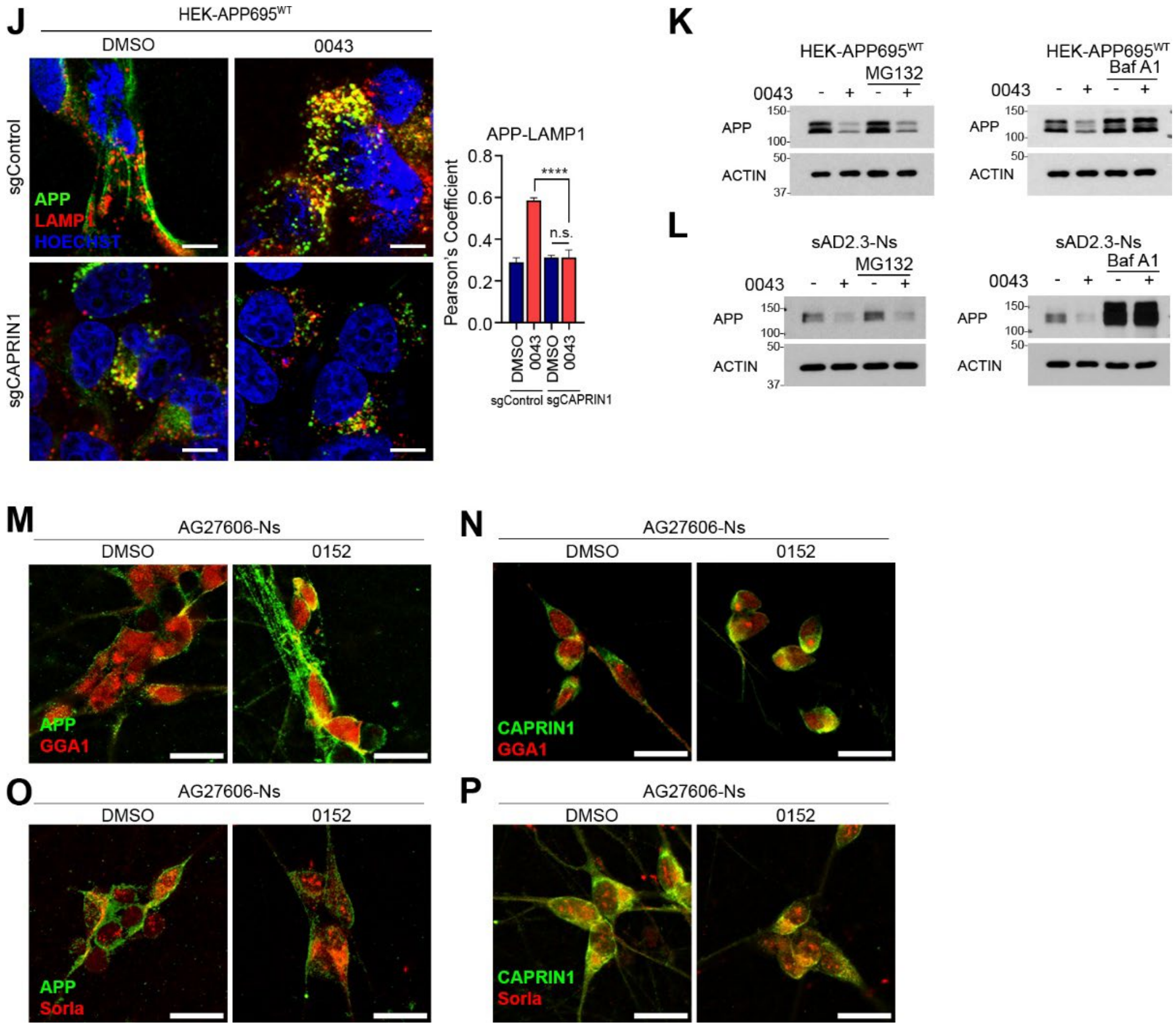

Figure. S5. Small-molecule glue degraders enhance CAPRIN1-dependent lysosomal degradation of APP in AD iPSC-derived neurons.

(**A**) Schematic of APP amyloidogenic and degradation pathways, highlighting CAPRIN1-mediated lysosomal degradation. (**B-G**) Time-course confocal images of APP (B–D) and CAPRIN1 (E–G) trafficking in AG27606-Ns treated with 0043 (10 µM) for 0, 3, or 7 h; co-stained with EEA1 (B,E), Rab7 (C,F), or LAMP1 (D,G) and Hoechst. The 0 hr time point represents untreated cells prior to compound exposure and serves as the baseline control. Quantification of colocalization shown (mean ± s.d.; one-way ANOVA; n.s., $^{**}p < 0.01$, $^{****}p < 0.0001$); scale bars, 20 µm. (**H,I**) APP-LAMP1 colocalization in sAD2.3 (H) and UKB011A neurons (I) treated with DMSO or 0043 (10 µM, 24 h) (mean ± s.d.; $^{*}p < 0.05$); scale bars, 20 µm. (**J**) Confocal images of sgControl and sgCAPRIN1 HEK-APP695^{WT} cells treated with DMSO or 0043 (10 µM, 24 h) stained for APP, LAMP1, and Hoechst (scale bar, 10 µm); APP-LAMP1 colocalization quantified (mean ± s.d., n = 3; n.s., $^{****}p < 0.0001$). (**K,L**) Western blots of APP in HEK-APP695^{WT} (K) or sAD2.3-Ns (L) pretreated with MG132 or Bafilomycin A1, followed by 0043 (10 µM, 24 h). (**M,N**) Confocal images of AG27606-Ns treated with DMSO or 0152 (10 µM, 24 h) stained for APP/GGA1 (m) or CAPRIN1/GGA1 (n); scale bars, 20 µm. (**O,P**) Confocal images of AG27606-Ns treated with DMSO or 0152 stained for APP/Sorla (o) or CAPRIN1/Sorla (p);– scale bars, 20 µm.

Figure S6

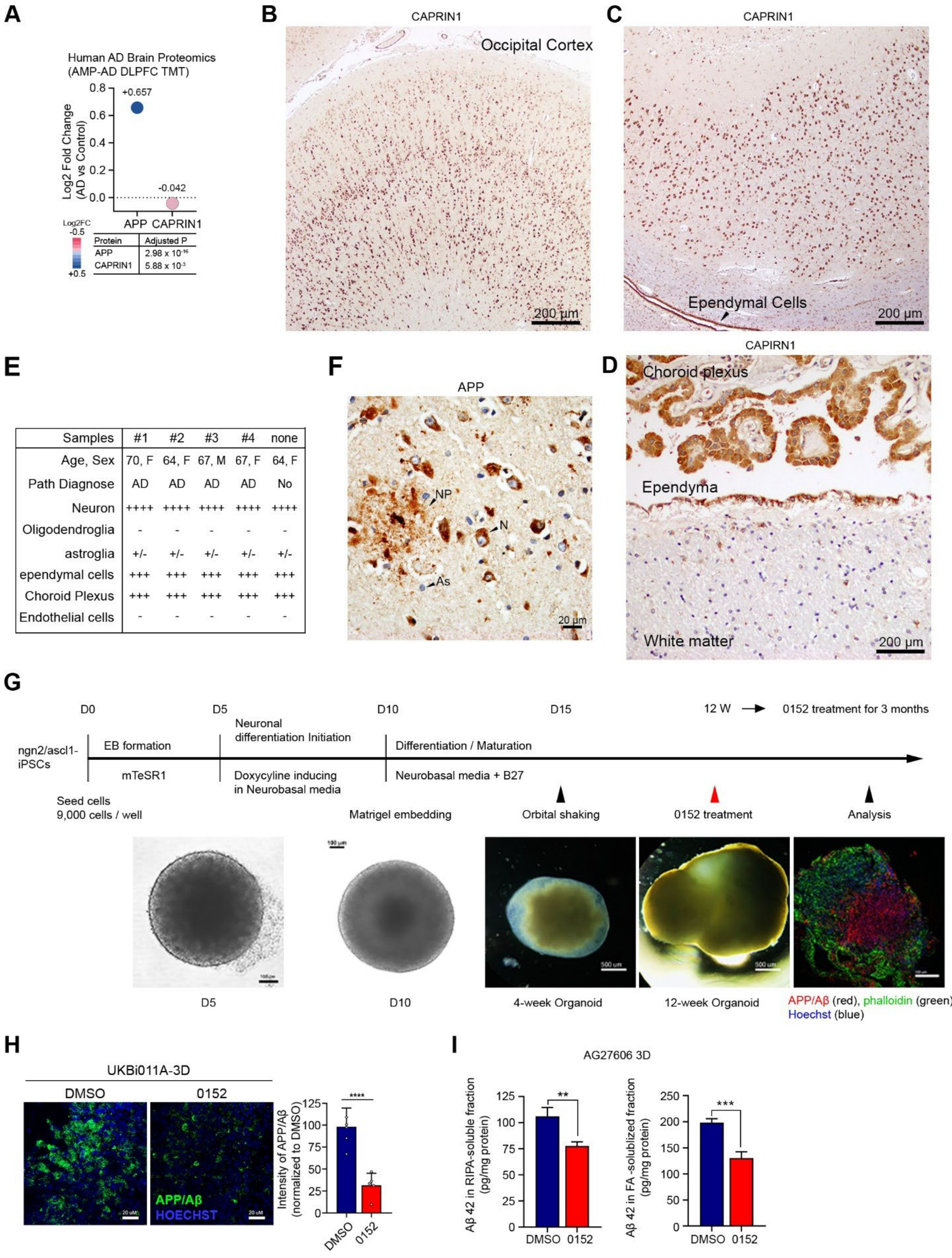

Samples	#1	#2	#3	#4	none
Age, Sex	70, F	64, F	67, M	67, F	64, F
Path Diagnose	AD	AD	AD	AD	No
Neuron	++++	++++	++++	++++	++++
Oligodendroglia	-	-	-	-	-
astroglia	+/-	+/-	+/-	+/-	+/-
ependymal cells	+++	+++	+++	+++	+++
Choroid Plexus	+++	+++	+++	+++	+++
Endothelial cells	-	-	-	-	-

Figure. S6. CAPRIN1 expression in human AD brains and 0152-mediated APP/Aβ reduction in iPSC-derived organoids.

(A) Protein abundance was analyzed using the AMP-AD genome-wide tandem mass tag (TMT) proteomic dataset from the dorsolateral prefrontal cortex (DLPFC), accessed through the AGORA platform. APP protein abundance was significantly increased in AD brains (approximately 58%; log2FC = 0.657, adjusted $P = 2.98 \times 10^{-16}$), whereas CAPRIN1 abundance exhibited only a minimal reduction (log2FC = -0.042, adjusted $P = 5.88 \times 10^{-3}$). Colors indicate the direction and magnitude of differential protein expression (log2FC). (**B-D**) IHC of CAPRIN1 in AD patient brain sections showing expression in occipital cortex (B), ependymal cells lining ventricles (C), and choroid plexus (D); scale bars, 200 µm. (**E**) Summary of pathological classification of AD and non-AD patient brain samples. (**F**), APP/Aβ IHC in cortex showing neuronal (N), astrocytic (As) and neuritic plaque (NP) labeling (scale bar, 20 µm). (**G**) Timeline and representative images of brain organoid generation from Ngn2/Ascl1-iPSCs: EB aggregation (5 days), doxycycline-induced neuronal differentiation (5 days), Matrigel embedding at D10, orbital shaking from D15, drug treatment at week 12, and representative organoid at week 16 stained for APP/Aβ (red), phalloidin (green), Hoechst (blue). (**H**) Confocal images and quantification of APP/Aβ intensity in UKBi011A-derived 3D organoids treated with DMSO or 0152 (10 µM) (scale bar, 20 µm; mean ± s.d.; $n \geq 4$). **(I)** Aβ42 levels in AG27606-derived 3D organoids treated with DMSO or 0152, measured by ELISA. Organoids were sequentially extracted into RIPA-soluble and Formic acid-solubilized fractions. Aβ42 levels were normalized to total protein (pg/mg protein). Data are mean ± s.d. ($n = 3$; $^{**}p < 0.01$, $^{***}p < 0.001$).

Figure S7

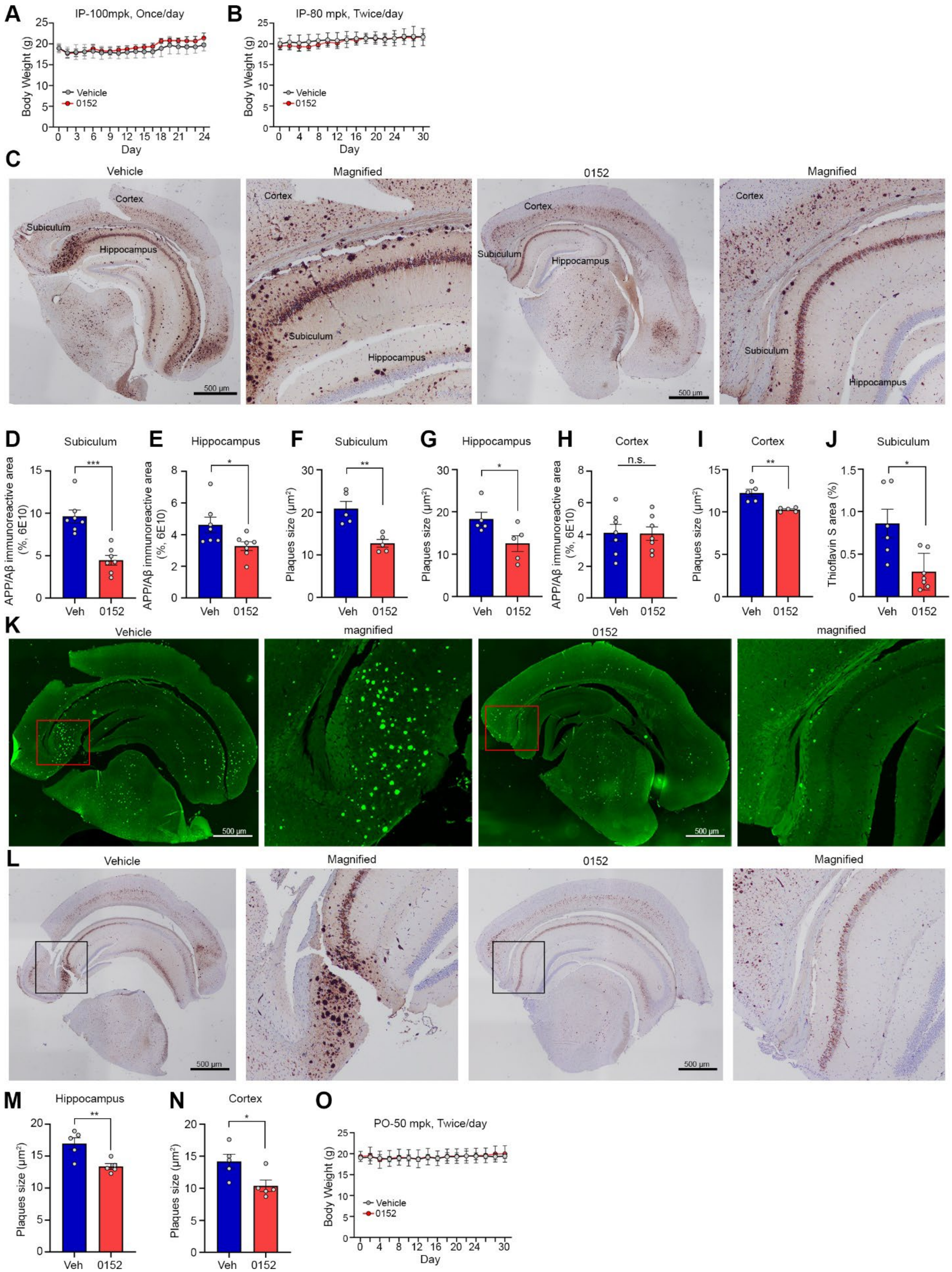

A
IP-100mpk, Once/day
Body Weight (g)
Vehicle
0152
Day
B
IP-80 mpk, Twice/day
Body Weight (g)
Vehicle
0152
Day
C
Vehicle
Magnified
0152
Magnified
Cortex
Subiculum
Hippocampus
500 μm
D
Subiculum
APP/Aβ immunoreactive area (%, 6E10)
***
Veh
0152
E
Hippocampus
*
F
Subiculum
Plaques size (μm²)
**
G
Hippocampus
*
H
Cortex
n.s.
I
Cortex
**
J
Subiculum
Thioflavin S area (%)
*
K
Vehicle
magnified
0152
magnified
L
Vehicle
Magnified
0152
Magnified
M
Hippocampus
**
N
Cortex
*
O
PO-50 mpk, Twice/day

Figure. S7. Systemic administration of 0152 reduce APP/ Aβ levels and amyloid burden in the brains of 5xFAD mice.

(**A,B**) Body weights during 0152 treatment at different dosing regimens: (A) IP once daily 100 mg/kg; (B) IP twice daily 80 mg/kg (mean ± s.e.m.). (**C**) IHC of 5xFAD mouse brains showing APP/Aβ accumulation in cortex, hippocampus, and subiculum after 30-day vehicle or 0152 treatment (80 mg/kg, IP twice); scale bars, 500 µm. (**D-I**) Quantification of APP/Aβ-positive area and plaque size in subiculum, hippocampus, and cortex (mean ± s.e.m.; *$p < 0.05$, **$p < 0.01$, ***$p < 0.001$; n.s.; unpaired two-tailed t-test). (**J,K**) Thioflavin S staining (J) and quantification of Thioflavin S-positive area in subiculum (K) after 0152 treatment (mean ± s.e.m.; *$p < 0.05$; unpaired two-tailed t-test). (**L**) IHC showing APP/Aβ accumulation after 30-day vehicle or 0152 treatment by oral gavage (80 mg/kg, PO twice); scale bars, 500 µm. (**M**,**N**) Quantification of APP/Aβ plaque size in hippocampus (m) and cortex (n) after oral treatment (mean ± s.e.m.; *$p < 0.05$, **$p < 0.01$). (**O**) Body weight monitoring during PO twice daily 80 mg/kg 0152 treatment (mean ± s.e.m.).

Table S1. Oligonucleotides

**CRISPR**		
Caprin1 (Oligo1)	IDT	5'-CACCGCGACAAGAAACTTCGGAACC-3'
Caprin1 (Oligo2)	IDT	5'AAACGGTTCCGAAGTTTCTTGTCG C-3'
Control primer	Addgene	80248
**qPCR primers**		
APP (Forward)	IDT	5′-TGACAAGTTCCGAGGGGTAG-3′
APP (Reverse)	IDT	5′-CCAGACATCCGAGTCATCCT-3′
GAPDH (Forward)	IDT	5′-CTCTGACTTCAACAGCAC-3′
GAPDH (Reverse)	IDT	5′-CATACCAGGAAATGAGCTTGACAA-3′

Table S2. Antibodies

Target	Clone	Host	Vendor	Catalog #	Application
Aβ (1–16)	6E10	Mouse	BioLegend	803001	WB, IF, IHC
Aβ (17–24) (Biotin)	4G8	Mouse	BioLegend	800704	IP
APP (C-terminal)		Rabbit	Sigma	A8717	WB, IP
APP (N-terminal)	22C11	Mouse	Millipore	MAB348	IF, IHC
APLP1		Rabbit	Proteintech	12305-2-AP	WB
APLP2		Rabbit	Proteintech	15041-1-AP	WB
Actin	AC-15	Mouse	Sigma	A1978	WB
CAPRIN1		Rabbit	Proteintech	15112-1-AP	WB, IF, IP, IHC
PSEN1		Rabbit	Cell Signaling Technology	5643	WB
PSEN2		Rabbit	Cell Signaling Technology	9979	WB
BACE1		Rabbit	Abcam	ab2077	WB
ADAM10		Rabbit	Cell Signaling Technology	14194	WB
G3BP1		Mouse	Proteintech	66486-1-Ig	WB
Nicastrin		Rabbit	Cell Signaling Technology	5665	WB
GGA1		Rabbit	Proteintech	25674-1-AP	WB, IF
SORLA		Rabbit	Proteintech	22592-1-AP	WB, IF
Neprilysin (CD10)		Rabbit	Cell Signaling Technology	65534	WB
LAMP1		Mouse	BioLegend	328601	WB
LAMP1	D2D11	Rabbit	Cell Signaling Technology	9091S	IF
TUBB3		Mouse	Proteintech	66375-1-Ig	IF
MAP2	AP18	Mouse	Thermo Fisher	MA5-12826	IF
SOX2		Rabbit	Proteintech	11064-1-AP	IF
NANOG		Rabbit	Thermo Fisher	PA1-097	IF
Calnexin		Rabbit	Proteintech	10427-2-AP	IF
ERGIC-53		Rabbit	Proteintech	13364-1-AP	IF
SEC31A		Rabbit	Proteintech	17913-1-AP	IF
TMED9		Rabbit	Proteintech	21620-1-AP	IF
GM130	D6B1	Rabbit	Cell Signaling Technology	12480S	IF
TGN46		Rabbit	Proteintech	13573-1-AP	IF
EEA1	C45B10	Rabbit	Cell Signaling Technology	3288S	IF
Rab7	D95F2	Rabbit	Cell Signaling Technology	9367S	IF
HiBiT		Mouse	Promega	N7200	WB, IP
Goat anti-mouse IgG (Alexa Fluor 488)		Goat	Invitrogen	A-11001	IF
Goat anti-rabbit IgG (Alexa Fluor 488)		Goat	Invitrogen	A-11008	IF

Goat anti-mouse IgG (Alexa Fluor 555)		Goat	Invitrogen	A-21422	IF
Goat anti-rabbit IgG (Alexa Fluor 555)		Goat	Invitrogen	A-21428	IF

Table S3. MDR1-MDCKII permeability and efflux data for compounds 0043 and 0152

Compound	Apical to basal (AP-BL) Papp ($X10^{-6}$cm/sec)	Basal to apical (BL-AP) Papp ($X10^{-6}$cm/sec)	Efflux ratio	% Recovery (Overall) - Apical to basal	% Recovery (Overall) - Basal to Apical	% CV (Overall) - Apical to basal	% CV (Overall) - Basal to Apical
0043	0.4	42.6	114	121.0	106.5	18.6	7.0
0152	0.5	37.4	78	120.9	141.3	22.2	0.8

Supplemental Materials And Methods

Synthesis of compound 0043

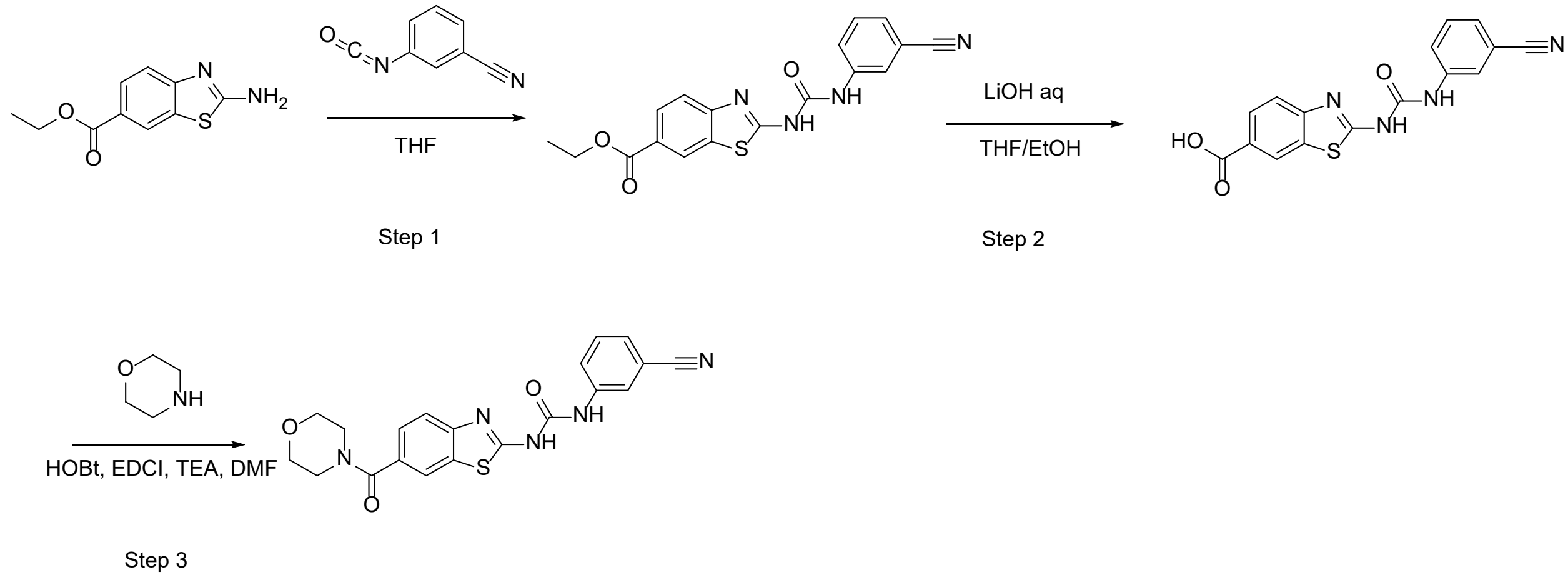

Step 1. Synthesis of methyl 2-[(3-cyanophenyl)carbamoylamino]-1,3-benzothiazole-6-carboxylate: Methyl 2-amino-1,3-benzthiazole-6-carboxylate (2 g, 9.6 mmol) was suspended in Tetrahydrofuran (60 mL) and reacted with 3-isocyanatobenzonitrile (1.65 g, 11.5 mmol) at 25°Cfor 16 hours. TLC was run in 1:1 Ethyl acetate/Hexane to show completion. The reaction mixture was concentrated then diluted with 40 mL of ethanol. After heating the reaction mixture to 70°C, 40 mL of dichloromethane was added and the reaction was stirred for 30 minutes. The solid was then cooled to 25°C, filtered, and rinsed with ethyl alcohol to give the title compound as a white solid (2.8 g, yield: 83%).

Step 2. Synthesis of 2-[(3-cyanophenyl)carbamoylamino]-1,3-benzothiazole-6-carboxylic acid: Methyl 2-[(3-cyanophenyl)carbamoylamino]-1,3-benzothiazole-6-carboxylate (2.8 g, 7.9 mmol) was suspended in a 2:1 solution of Tetrahydrofuran / Ethyl alcohol (80 mL). An aqueous 1N Lithium hydrate solution (58.1 mmol) was added to the reaction and stirred at 25°C for 2 days. TLC in 1:1 Ethyl acetate/Hexane showed completion. The reaction was concentrated to remove the organic solvents, and the residue was diluted with 20 mL of H_2O and 80 mL of Isopropyl alcohol. The pH was adjusted to 5 using Acetic acid and after stirring for 1 hour, the reaction was filtered and rinsed with Isopropyl alcohol to obtain the title compound as a white solid (2.3 g, yield: 88%).

Step 3. Synthesis of 0043: 2-[(3-cyanophenyl)carbamoylamino]-1,3-benzothiazole-6-carboxylic acid (2.3 g, 6.8 mmol) was suspended in DMF (90 mL), then EDCI (2.1 g, 13.6 mmol) and HOBt (1.1 g, 8.16 mmol) were added to the reaction. After stirring at 25°Cfor 10 minutes, Morpholine (2.9 g, 34 mmol) and Triethylamine (9.4 mL, 68 mmol) was added, then the reaction for stirred for 2 days at 25°C. TLC in 1:1 Ethyl acetate/Hexane showed completion, the reaction was poured into 200 mL of H_2O and washed three times with 200 mL of Ethyl acetate. The organic washes were combined and concentrated to ~100 mL, and 100 mL of H_2O was added. The organic and aqueous were left alone for 8 hours until a white solid precipitated out of the solution. The solid was then filtered and rinsed with H_2O to give the title compound 0043 (1.2 g, yield: 43%) as a white solid.

Synthesis of compound 0152

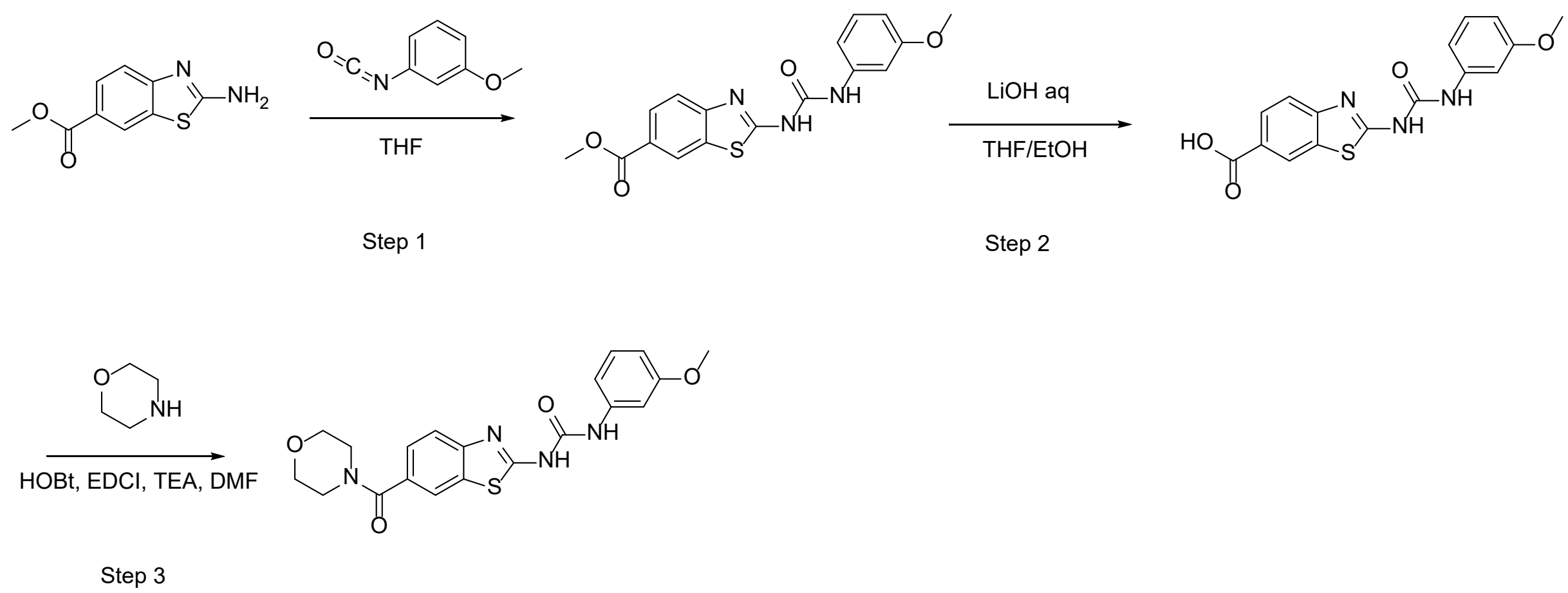

Step 1. Synthesis of methyl 2-(3-(3-methoxyphenyl)ureido)benzo[d]thiazole-6-carboxylate: Methyl 2-amino-1,3-benzthiazole-6-carboxylate (2 g, 9.6 mmol) was suspended in THF (60 mL) and reacted with 1-methoxy-3-isocyanato-benzene (1.72 g, 11.5 mmol) at 25°C for 16 hours. TLC was run in 1:1 Ethyl acetate/Hexane to show completion. The reaction was concentrated then diluted with 40 mL of Ethyl alcohol. After heating the reaction mixture to 70°C, 40 mL of Dichloromethane was added and the reaction was stirred for 30 minutes. The solid was then cooled to 25°C, filtered and rinsed with Ethyl alcohol to give the title compound as a white solid (2.6 g, yield: 76%).

Step 2. Synthesis of 2-(3-(3-methoxyphenyl)ureido)benzo[d]thiazole-6-carboxylic acid: Methyl 2-(3-(3-methoxyphenyl)ureido)benzo[d]thiazole-6-carboxylate (2.6 g, 7.3 mmol) was suspended in a 2:1 solution of Tetrahydrofuran / Ethyl alcohol (80 mL). An aqueous 1N Lithium hydrate solution (58.1 mmol) was added to the reaction and stirred at 25°C for 2 days. TLC in 1:1 Ethyl acetate/Hexane showed completion. The reaction was concentrated to remove the organic solvents and was diluted with 20 mL of H_2O and 80 mL of Isopropyl alcohol. The pH was adjusted to 5 using Acetic acid, and after stirring for 1 hour, the reaction was filtered and rinsed with Isopropyl alcohol to give the title compound as a white solid (2.4 g, yield: 97%).

Step 3. Synthesis of 0152: 2-(3-(3-methoxyphenyl)ureido)benzo[d]thiazole-6-carboxylic acid (2.4 g, 7.3 mmol) was suspended in DMF (90 mL), then EDCI (2.8 g, 14.6 mmol) and HOBt (1.19 g, 8.78 mmol) was added to the reaction. After stirring at 25°C for 10 minutes, morpholine (2.55 g, 29.3 mmol) and triethylamine (8.21 mL, 58.5 mmol) were added, then the reaction for stirred for 2 days at 25°C. TLC in 1:1 Ethyl acetate/Hexane showed completion, the reaction was poured into 200 mL of H_2O and washed three times with 200 mL of Ethyl acetate. The organic washes were combined and concentrated to ~100 mL, and 100 mL of H_2O was added. The organic and aqueous were left alone for 8 hours until a white solid precipitated out of solution. The solid was then filtered and rinsed with H_2O to give 0152 as a white solid (800 mg, yield: 27%).

Physicochemical Property Calculations

Physicochemical properties including molecular weight (MW), calculated partition coefficient (cLogP), and polar surface area (PSA), as well as central nervous system multiparameter optimization (CNS MPO) scores, were calculated using the Schrödinger LiveDesign platform.

CNS MPO scores were derived based on standard multiparameter optimization criteria incorporating cLogP, PSA, MW, hydrogen bond donors, and pKa.

Recombinant protein purification from E. coli

For CAPRIN1 purification, pET-6HIS-TEV-hCAPRIN1 (Vectorbuilder) were transformed into E. coli BL21 (DE3) followed by culturing in Luria broth (LB) with 100 μg/mL of Ampicillin overnight at 37°C. 10 ml of primary culture was inoculated into fresh LB medium with Ampicillin, cultured until OD_{600} reached 0.8, and expression of CAPRIN1 was induced with 0.5 mM isopropyl-β-D-thiogalactoside at 37°C for 4 hours. After centrifugation at 3,000 rpm for 20 minutes at 4°C, pellets were resuspended with prechilled lysis buffer (50 mM Na_2HPO_4, 300 mM NaCl, 10% Glycerol, 0.1% Triton X-100, 10 mM Imidazole at pH 8.0), followed by sonication for 16 minutes (10 seconds on and 30 seconds off for 24 cycles) on ice and centrifuged for 40 minutes (40,000 rpm at 4°C). The supernatant was mixed with Ni-NTA agarose beads (Qiagen) overnight at 4°C, then loaded on a PD-10 column (GE healthcare). The columns were washed with buffer (50 mM Na_2HPO_4, 300 mM NaCl, 10% Glycerol, 10 mM Imidazole at pH 8.0) and eluted with elution buffer (50 mM Na_2HPO_4, 300 mM NaCl, 10% Glycerol, 250 mM Imidazole at pH 8.0), followed by dialysis (50 mM Na_2HPO_4, 250 mM NaCl, 10% Glycerol at pH 8.0) for 2 hours at 4°C. The samples were concentrated using Millipore Amicon filters (Millipore) and dialyzed with storage buffer (50 mM Na_2HPO_4, 200 mM NaCl, 50% Glycerol at pH 8.0) for storage at -80°C.

Recombinant protein from insect cells

The DNA cassette (10xHis-3C site or 10xHis-MBP-3C site) was inserted immediately 5' of the *Bam*HI site in the pFastBac vector (Invitrogen, 10360014) using the SLIC method, yielding constructs termed pSEP0(10His-3C) (pYT1353), or pSEP8 (10His-MBP-3C) (pYT1566) vector, respectively. The open reading frame (ORF) of human CAPRIN1 was codon-optimized, synthesized, and sub-cloned into the BamHI and HindIII site of a pSEP0 vector, yielding pSEP0-CAPRIN1 (10xHis-3C-CAPRIN1) (pYT1837) vector. The open reading frame (ORF) of a full-length human APP (APP695) was codon-optimized, synthesized, and sub-cloned into the BamHI and HindIII site of a pSEP8 vector, yielding pSEP8-APP695 (10xHis-MBP-3C-APP695) (pYT1842) vector. Generation of baculovirus expressing 10xHis-CAPRIN1 or 10xHis-MBP-APP695 using pSEP0-CAPRIN1 or pSEP8-APP695 vector in Sf9 cells (Expression Systems, Inc) was performed as described previously. Expression of recombinant CAPRIN1, or APP695 was optimized using the TEQC method in which a 200 ml culture of Hi5 cells (Expression Systems, Inc, 94-002S) was infected at an estimated multiplicity of infection (eMOI) of 4. After a 96-hour incubation at 27°C, cells were harvested, frozen in liquid nitrogen and kept at -80°C until use.

For the purification of CAPRIN1, cell pellet was lysed in 50 mM Hepes-KOH pH 7.6, 400 mM potassium chloride (KCl), 10 % Glycerol, 5 mM β-mercaptoethanol, 10 mM Imidazole, and 0.5 ml of 100X protease cocktail (6 mM Leupeptin, 0.2 mM Pepstatin A, 20 mM Benzamidine, and 10 mM PMSF). Cell lysate was stirred for 30 min followed by centrifugation at 100,000 g for 45 min. The supernatant was loaded onto Ni-resin (HIS-Select, Sigma-Aldrich) and washed with buffer 1 (50 mM Hepes-KOH pH7.6, 1 M KCl, 5% Glycerol, 10 mM Imidazole, and 5 mM β-mercaptoethanol), and buffer 2 (50 mM Hepes-KOH pH7.6, 2 M KCl, 5% Glycerol, 10 mM Imidazole, and 5 mM β-mercaptoethanol) followed by lysis buffer and the low salt buffer (50 mM Hepes-KOH pH7.6, 200 mM KCl, 5% Glycerol, 10 mM Imidazole, and 5 mM β-mercaptoethanol). 10xHis-CAPRIN1 was eluted from Ni-resin with the elution buffer (50 mM Hepes-KOH pH7.6, 200 mM potassium acetate, 5% Glycerol, 300 mM Imidazole, and 5 mM β-mercaptoethanol).

Elution fractions containing 10xHis-CAPRIN1 were pooled and dialyzed overnight against the low salt buffer. Dialysate was concentrated by spin column (Amicon 10k cut-off) to 4.0 mg/ml. Recombinant CAPRIN1 was further purified by 10–30% (v/v) glycerol gradient containing the buffer (50 mM Hepes-KOH pH7.6, 200 mM KCl, 5% glycerol and 5 mM β-mercaptoethanol). After centrifugation at 35,000 rpm in a Beckman SW Ti-40 rotor for 19 hours at 4°C, the gradient was fractionated using a PGF Piston Gradient Fractionator (BioComp Instruments). Fractions containing 10xHis-CAPRIN1 were pooled and dialyzed overnight against the low salt buffer. Dialysate was concentrated by spin column (Amicon 10k cut-off) to 4 mg/ml. Concentration of purified protein was measured by Bradford method. When purifying APP695, the same procedure as the CAPRIN1 purification was used. Buffer 1 (50 mM Hepes-KOH pH7.6, 1 M KCl, 5% Glycerol, 5 mM β-mercaptoethanol, and 0.1% DDM), wash buffer 2 (50 mM Hepes-KOH pH7.6, 2 M KCl, 5% Glycerol, 5 mM β-mercaptoethanol, and 0.1% DDM), low salt buffer with 0.1% DDM, elution buffer (50 mM Hepes-KOH pH7.6, 200 mM potassium acetate, 5% Glycerol, 50 mM Maltose, and 5 mM β-mercaptoethanol), and Amylose-resin were used.
